# HDAC 1/4-mediated silencing of microRNA-200b promotes chemoresistance in human lung adenocarcinoma cells

**DOI:** 10.18632/oncotarget.1948

**Published:** 2014-05-07

**Authors:** Dong-Qin Chen, Ban-Zhou Pan, Jia-Yuan Huang, Kai Zhang, Shi-Yun Cui, Wei De, Rui Wang, Long-Bang Chen

**Affiliations:** ^1^ Department of Medical Oncology, Jinling Hospital, School of Medicine, Nanjing University, Nanjing, Jiangsu 210002, China; ^2^ Department of Biochemistry and Molecular Biology, Nanjing Medical University, Nanjing, Jiangsu 210002, China

**Keywords:** lung adenocarcinoma, miR-200b, E2F3, histone deacetylase, chemoresistance

## Abstract

Chemoresistance is one of the most significant obstacles in lung adenocarcinoma (LAD) treatment, and this process involves genetic and epigenetic dysregulation of chemoresistance-related genes. Previously, we have shown that restoration of microRNA (miR)-200b significantly reverses chemoresistance of human LAD cells by targeting E2F3. However, the molecular mechanisms involved in the silencing of miR-200b are still unclear. Here we showed that histone deacetylase (HDAC) inhibitors could restore the expression of miR-200b and reverse chemoresistant phenotypes of docetaxel-resistant LAD cells. HDAC1/4 repression significantly increased miR-200b expression by upregulating histone-H3 acetylation level at the two miR-200b promoters partially via a Sp1-dependent pathway. Furthermore, silencing of HDAC1/4 suppressed cell proliferation, promoted cell apoptosis, induced G_2_/M cell cycle arrest and ultimately reversed *in vitro* and *in vivo* chemoresistance of docetaxel-resistant LAD cells, at least partially in a miR-200b-dependent manner. HDAC1/4 suppression-induced rescue of miR-200b contributed to downregulation of E2F3, survivin and Aurora-A, and upregulation of cleaved-caspase-3. HDAC1/4 levels in docetaxel-insensitive human LAD tissues, inversely correlated with miR-200b, were upregulated compared with docetaxel-sensitive tissues. Taken together, our findings suggest that the HDAC1/4/Sp1/miR-200b/E2F3 pathway is responsible for chemoresistance of docetaxel-resistant LAD cells.

## INTRODUCTION

Lung cancer, a predominant public health problem worldwide, is responsible for more cancer-related deaths than any other cancer in both women and men [1]. Lung cancer was expected to account for 28% of all male cancer deaths and 26% of all female cancer deaths in 2013 [2]. Lung adenocarcinoma (LAD) is the most common histological form of lung cancer and chemotherapy is a significant component of the current first-line therapies for LAD. However, chemoresistance has become a major obstacle in chemotherapeutic treatment of LAD, especially at advanced stage, and is associated with poor prognosis of patients. Therefore, identification of the underlying molecular mechanisms of chemoresistance in LAD has become a key issue in clinical treatment of human LAD.

MicroRNAs (miRNAs) are short noncoding RNAs that silence their target mRNAs by binding to the 3′-untranslated region of target genes and function as important post-transcriptional regulators of chemoresistance-associated genes [3-7]. Recently, miRNAs have been reported to be associated with chemoresistance of cancer, mainly through abnormal regulation of cell viability [8], cell apoptosis [9], cell cycle distribution [10], epithelial mesenchymal transition (EMT) [11] and cancer stem cell self-renewal [12, 13]. The miR-200 family members, consisting of miR-200b, miR-200c, miR-429, miR-200a and miR-141, are considered tumor suppressor miRNAs that can differentially regulate cell viability, invasion, apoptosis, cell cycle progression and EMT during tumor development and progression [14-16]. MiR-200b, a potent tumor suppressor located in the miR-200b/c/429 gene cluster, has already been linked to chemoresistance of tumor cells by modulating cell viability, cell apoptosis, EMT and cancer stem cell self-renewal [13, 17].

Previously, we have showed that miR-200b is significantly downregulated in docetaxel-resistant LAD cells, and restoration of miR-200b can enhance their *in vitro* and *in vivo* chemosensitivity by post-transcriptionally downregulating E2F3 [18]. Nevertheless, the underlying molecular mechanisms responsible for downregulation of miR-200b in docetaxel-resistant LAD cells have not been fully illustrated. Epigenetic gene repression has been regarded as a hallmark of tumor cells and plays pivotal roles in silencing of tumor suppressors. DNA methylation and histone modification are the two major forms of epigenetic changes at the promoter regions of genes. Several studies have demonstrated that DNA methylation is one of the potential molecular mechanisms controlling miR-200b expression in tumor cells [19, 20]. DNA hypermethylation-mediated silencing of miR-200b has been reported to be associated with cancer progression in primary lung tumors and advanced breast cancer [19, 20]. However, administration of a universal DNA methyltransferase inhibitor (5-aza-2'-deoxycytidine, 5-aza-dC) has no obvious effects on miR-200b expression in docetaxel-resistant LAD cells, suggesting that DNA methylation might not be the specific mechanism underlying downregulation of miR-200b in docetaxel-resistant LAD cells.

Histone acetylation, modulated by histone deacetylases (HDACs) and histone acetyltransferases (HATs) [21], represents one of the most important epigenetic mechanisms controlling gene expression and regulates various pathological processes by the transcriptional regulation of genes [22]. HDACs are a class of chromatin-modulating enzymes that remove acetyl groups from lysine residues around gene promoters and play a crucial role in epigenetic regulation of gene expression [23-25]. Recently, several studies indicated that inhibition of HDACs contributes to the upregulation of miRNAs involved in several cancers [23, 26, 27]. HDAC repression-induced overexpression of miR-15a, miR-16 and miR-29b is associated with induction of cell death in primary chronic lymphocytic leukemia cells [23]. HDAC1–3 inhibition-mediated miR-449 expression promotes cell apoptosis and reduces cell proliferation in hepatocellular carcinoma [27]. The HDAC4/Sp1/miR-200a regulatory network responsible for the downregulation of miR-200a enhances the proliferation and migration of hepatocellular carcinoma cells [28]. Together this suggests that histone acetylation might be an important molecular mechanism involved in regulation of miR-200b in docetaxel-resistant LAD cells.

The purpose of this study was to investigate the possible molecular mechanisms by which HDACs negatively regulate miRNA-200b expression and elucidate the relationship between histone acetylation-modulated miR-200b expression and chemoresistance of LAD cells. To the best of our knowledge, there have been no reports about HDAC-mediated silencing of miR-200b in regulating chemoresistance of LAD cells, and thus the present study could provide a novel strategy for reversing chemoresistance of LAD.

## RESULTS

### Histone deacetylase inhibitors elevate miR-200b levels and reverse chemoresistance of docetaxel-resistant LAD cells

**Figure 1:**
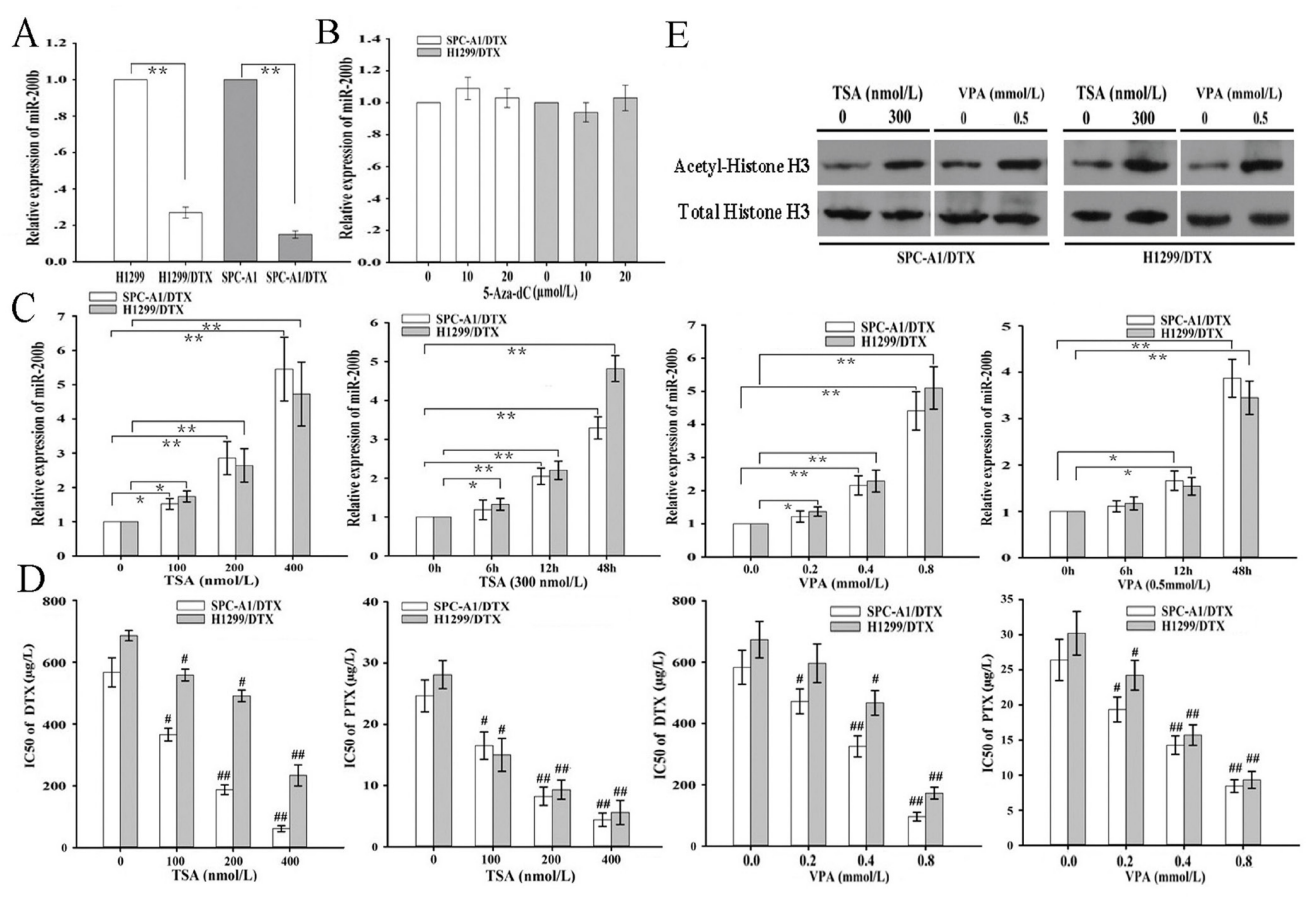
Histone deacetylase inhibitors (Trichostatin A, TSA, and valproic acid, VPA) elevate the expression of miR-200b and reverse chemoresistance of docetaxel-resistant LAD cells (A) qRT-PCR detection of relative miR-200b expression in docetaxel-resistant LAD cells (SPC-A1/DTX and H1299/DTX) and their parental LAD cells (SPC-A1 and H1299). Data were normalized to U6 RNA. (B) qRT-PCR detection of relative miR-200b expression in SPCA-1/DTX and H1299/DTX cells after treatment with DNA methyltransferase inhibitor (5-aza-2'-deoxycytidine, 5-aza-dC). Data were normalized to U6 RNA and determined relative to 0 µmol/L group. (C) qRT-PCR detection of relative miR-200b expression in SPCA-1/DTX and H1299/DTX cells after treatment with histone deacetylase inhibitor (TSA or VPA). Data were normalized to U6 RNA and determined relative to 0 µmol/L group. (D) MTT analysis of the IC50 values for docetaxel or paclitaxel in SPC-A1/DTX and H1299/DTX cells after treatment with TSA or VPA. (E) Western blot analysis of acetyl-histone H3 protein expression in SPC-A1/DTX and H1299/DTX cells after treatment with TSA or VPA. Data were the means ± standard error of at least three independent experiments. *P < 0.05, **P < 0.01; ^#^*P* < 0.05, ^##^*P* < 0.01 *vs* 0 nmol/L or 0 mmol/L group.

To better understand the underlying molecular mechanisms of chemoresistance in LAD, docetaxel-resistant LAD cell lines generated from parental H1299 or SPC-A1 cell lines were previously established in our lab. The miR-200b level as measured by qRT-PCR was significantly decreased in H1299/DTX and SPC-A1/DTX cell lines compared with that in H1299 and SPC-A1 cell lines, respectively (Figure 1A). To examine the potential epigenetic mechanisms responsible for downregulation of miR-200b in docetaxel-resistant LAD cells, histone deacetylase inhibitors (Trichostatin A, TSA, and valproic acid, VPA) or DNA methyltransferase inhibitor (5-aza-2'-deoxycytidine, 5-aza-dC) were administered to H1299/DTX and SPC-A1/DTX cells. Treatment of TSA and VPA significantly upregulated miR-200b expression, compared to no effects with 5-aza-dC, which indicated that histone acetylation might be the potential epigenetic mechanism responsible for downregulation of miR-200b levels in docetaxel-resistant LAD cells (Figure 1C, 1B). Acetyl-histone H3 protein levels in H1299/DTX and SPC-A1/DTX increased after treatment with TSA and VPA (Figure 1E). We next examined the effects of TSA and VPA on the IC_50_ values of docetaxel and paclitaxel in H1299/DTX and SPC-A1/DTX cells.It was showed that treatment of TSA and VPA could lead to the decreased IC_50_ values (docetaxel and paclitaxel) in H1299/DTX and SPC-A1/DTX cells in a dose-dependent manner (Figure 1D).

### HDAC1/Sp1 and HDAC4/Sp1 involvement in the downregulation of miR-200b in docetaxel-resistant LAD cells

**Figure 2:**
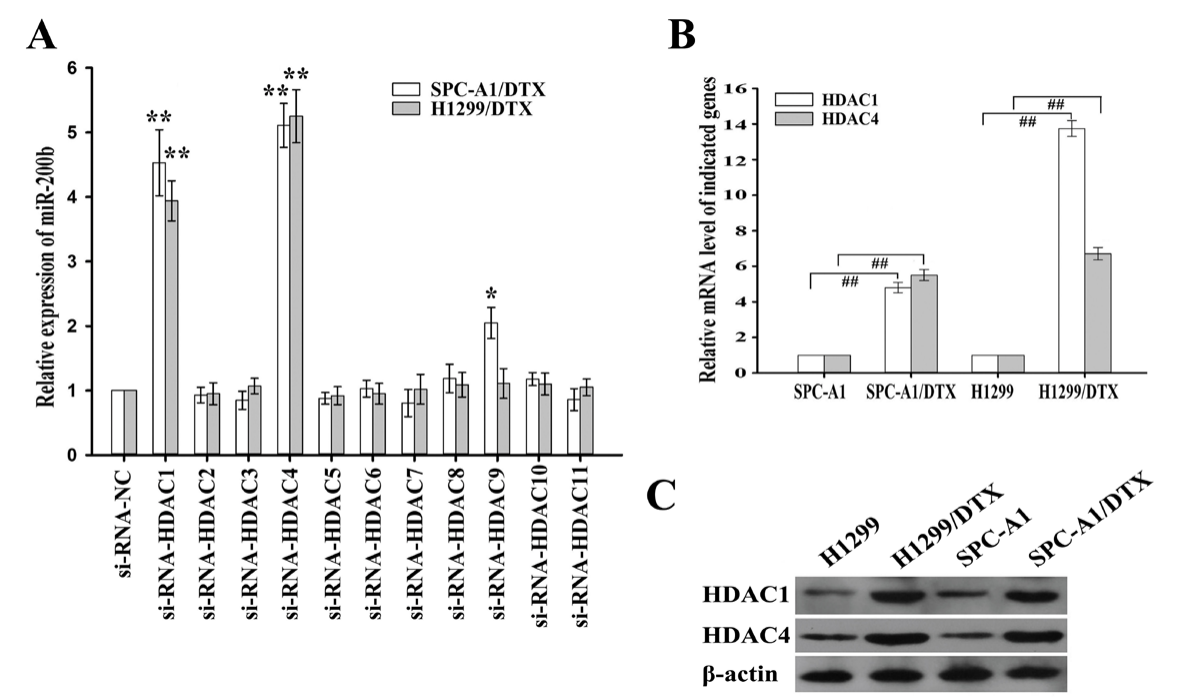
Effects of histone deacetylases (HDACs) on miR-200b expression (A) Relative levels of miR-200b as measured by qRT-PCR after transfection of the indicated siRNA-HDACs or siRNA-NC into H1299/DTX or SPC-A1/DTX cells. Data were normalized to U6 RNA. (B) Relative levels of HDAC1 or HDAC4 mRNA as measured by qRT-PCR in parental or docetaxel-resistant LAD cells. Data were normalized to GAPDH. (C) Western blot detection of HDAC1 or HDAC4 protein expression in parental or docetaxel-resistant LAD cells. β-actin was used as an internal control. Data were the means ± standard error of at least three independent experiments. ^##^*P* < 0.01; *P < 0.05, **P < 0.01 *vs* control.

To determine the specific HDACs involved in the regulation of miR-200b expression, vectors containing siRNAs against each of the eleven isoforms of HDACs were transfected into H1299/DTX and SPC-A1/DTX cells. 48 h after transfection, western blot confirmed the inhibitory efficiency of all HDACs-siRNA vectors (Supplementary Figure 1A). Notably, inhibition of HDAC1 or HDAC4 significantly upregulated the levels of miR-200b (Figure 2A). Results from qRT-PCR and western blot assays demonstrated that the expression levels of HDAC1 and HDAC4 mRNA and protein were significantly elevated in H1299/DTX and SPC-A1/DTX cells compared with those in parental H1299 and SPC-A1 cells, respectively (Figure 2B, 2C).

**Figure 3:**
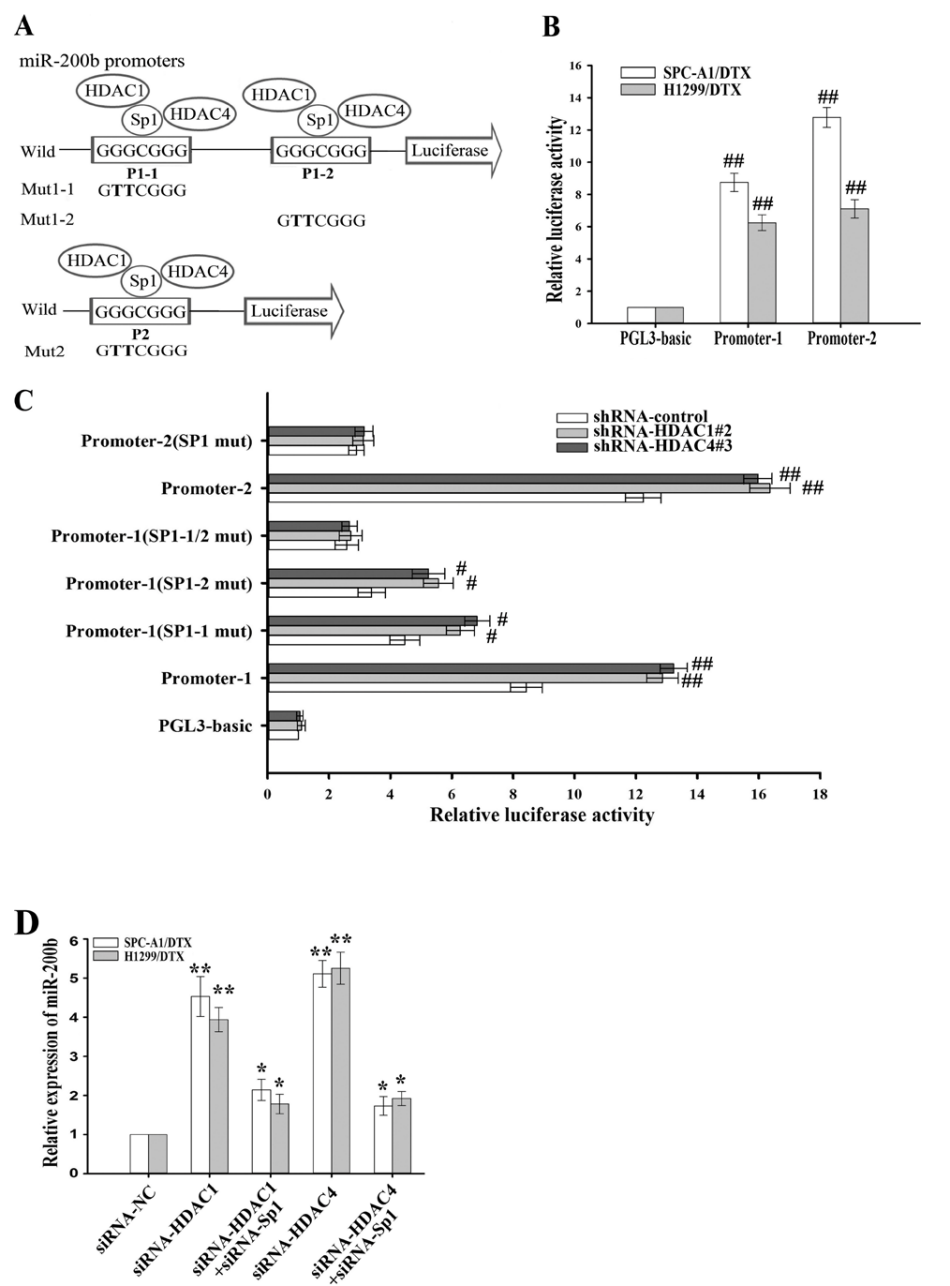
HDAC1/4 decreases miR-200b promoter activities (A) Schematic representation of the human miR-200b promoters with the putative Sp1-binding sites and the sequence of the point mutation. (B) Luciferase activity of docetaxel-resistant LAD cells cotransfected with Renilla and either wild-type miR-200b promoter or empty vector firefly reporter construct. Data were normalized to Renilla luciferase activity and determined relative to empty vector promoter activity. (C) Luciferase activity after transfection of sh-HDAC1#2, sh-HDAC4#3 or shRNA-control vector into SPC-A1/DTX cells co-transfected with Renilla and either wild-type miR-200b promoter, Sp1-binding mutant or empty vector firefly promoter reporter construct. Data were normalized to Renilla luciferase activity and determined relative to empty vector promoter activity. (D) qRT-PCR detection of relative miR-200b expression in H1299/DTX or SPC-A1/DTX cells transfected with siRNA-HDAC1, siRNA-HDAC4 or siRNA-NC or co-transfected with siRNA-HDAC1 or HDAC4 and siRNA-Sp1. Data were normalized to U6 RNA and determined relative to control group. Data were the means ± standard error of at least three independent experiments. ^#^*P* < 0.05, ^##^*P* < 0.01, *P < 0.05, **P < 0.01 *vs* control.

**Figure 4:**
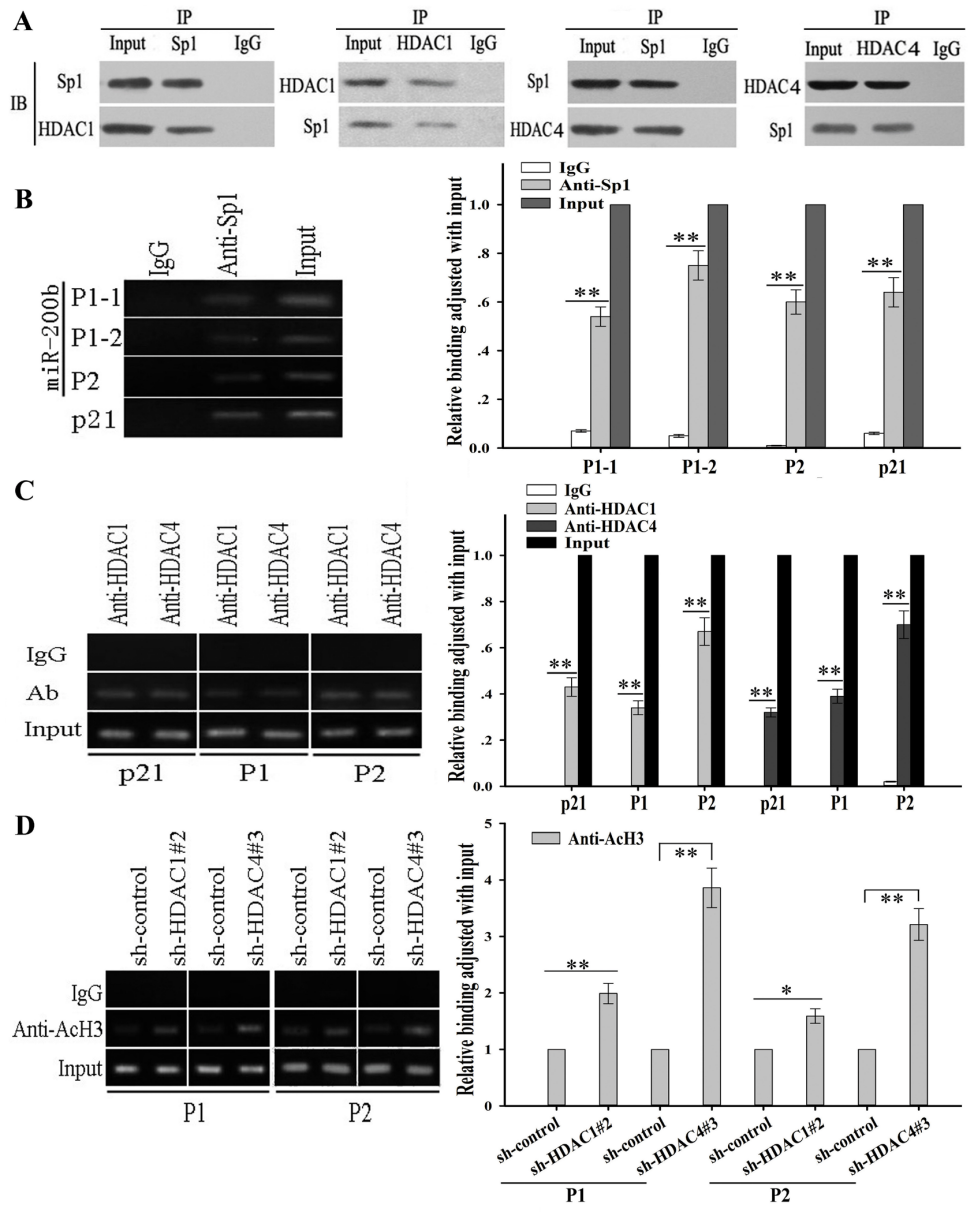
HDAC1/4 repression upregulates histone-H3 acetylation level at miR-200b promoters partially through the Sp1-dependent pathway (A) HDAC1/4 co-immunoprecipitates with Sp1 *in vivo* in SPC-A1/DTX cells. The levels of HDAC1/4 and Sp1 proteins were evaluated by western blot. (B) Sp1 and (C) HDAC1/4 binds to the miR-200b promoters *in vivo*. ChIP assays were performed in SPC-A1/DTX cells with antibodies against Sp1, HDAC1/4 or IgG control. Immunoprecipitated DNA was amplified by qRT-PCR with primers designed to amplify the sequences containing the putative Sp1-binding sites. Data are shown relative to qRT-PCR products amplified with input DNA before immunoprecipitation. p21 was used as a positive control. (D) HDAC1/4 decreases the histone-H3 acetylation level at the miR-200b promoters. ChIP assays were performed with antibody against acetyl-histone H3 in SPC-A1/DTX cells transfected with sh-control, sh-HDAC1#2 or sh-HDAC4#3. Immunoprecipitated DNA was amplified by qRT-PCR with primers designed to amplify the sequences containing the putative Sp1-binding sites. Data was normalized to qRT-PCR products amplified with input DNA before immunoprecipitation. Data were the means ± standard error of at least three independent experiments. *P < 0.05, **P < 0.01.

To further reveal the potential molecular mechanisms involved in transcriptional regulation of miR-200b, we analyzed the core regions of its promoters. Multiple Sp1 binding sites in the promoter regions could be identified, suggesting that Sp1 might be a key transcription factor of miR-200b. Schematics of the human miR-200b promoters are presented in Figure 3A and the putative Sp1-binding sites and mutations are indicated. The two promoters of the miR-200b gene were cloned into the pGL3 basic firefly luciferase reporter and evaluated in luciferase assays. Our data indicated that both promoters were active in H1299/DTX and SPC-A1/DTX cell lines (Figure 3B). To determine the effects of HDAC1 and HDAC4 in docetaxel-resistant LAD cells, several sh-RNA vectors of HDAC1 and HDAC4 were first transfected into H1299/DTX and SPC-A1/DTX cell lines. The sh-HDAC1#2 and the sh-HDAC4#3 were found as the most efficient shRNAs (Supplementary Figure 1B). Our results showed that suppression of HDAC1 and HDAC4 significantly increased luciferase activities of the two miR-200b luciferase promoter constructs (Figure 3C). However, when the Sp1-binding sites were mutated, the effects of HDAC1 and HDAC4 on miR-200b promoters were attenuated (Figure 3C). When H1299/DTX or SPC-A1/DTX cells were co-transfected with siRNA-HDAC1 or siRNA-HDAC4 and siRNA-Sp1, the upregulation of miR-200b induced by HDAC1/4 downregulation could be partially abrogated by Sp1 (Figure 3D). To determine whether HDAC1 or HDAC4 interacts with Sp1, we conducted co-immunoprecipitation analysis, and showed that HDAC1 and HDAC4 were associated with Sp1 *in vivo* (Figure 4A). Furthermore, chromatin immunoprecipitation (ChIP) assay showed that both HDAC1/4 and Sp1 co-localized to the miR-200b promoters at the Sp1-binding sites *in vivo* (p21 was used as a positive control) (Figure 4B, 4C). ChIP assay also revealed that inhibition of HDAC1/4 increased the histone-H3 acetylation level of the miR-200b promoters at Sp1-binding sites (Figure 4D). Therefore, the HDAC1/4-Sp1 complexes negatively regulate miR-200b expression in docetaxel-resistant LAD cells.

### HDAC1/4 repression reverses the *in vitro* chemoresistance of LAD cells partially in a miR-200b-dependent manner

**Figure 5:**
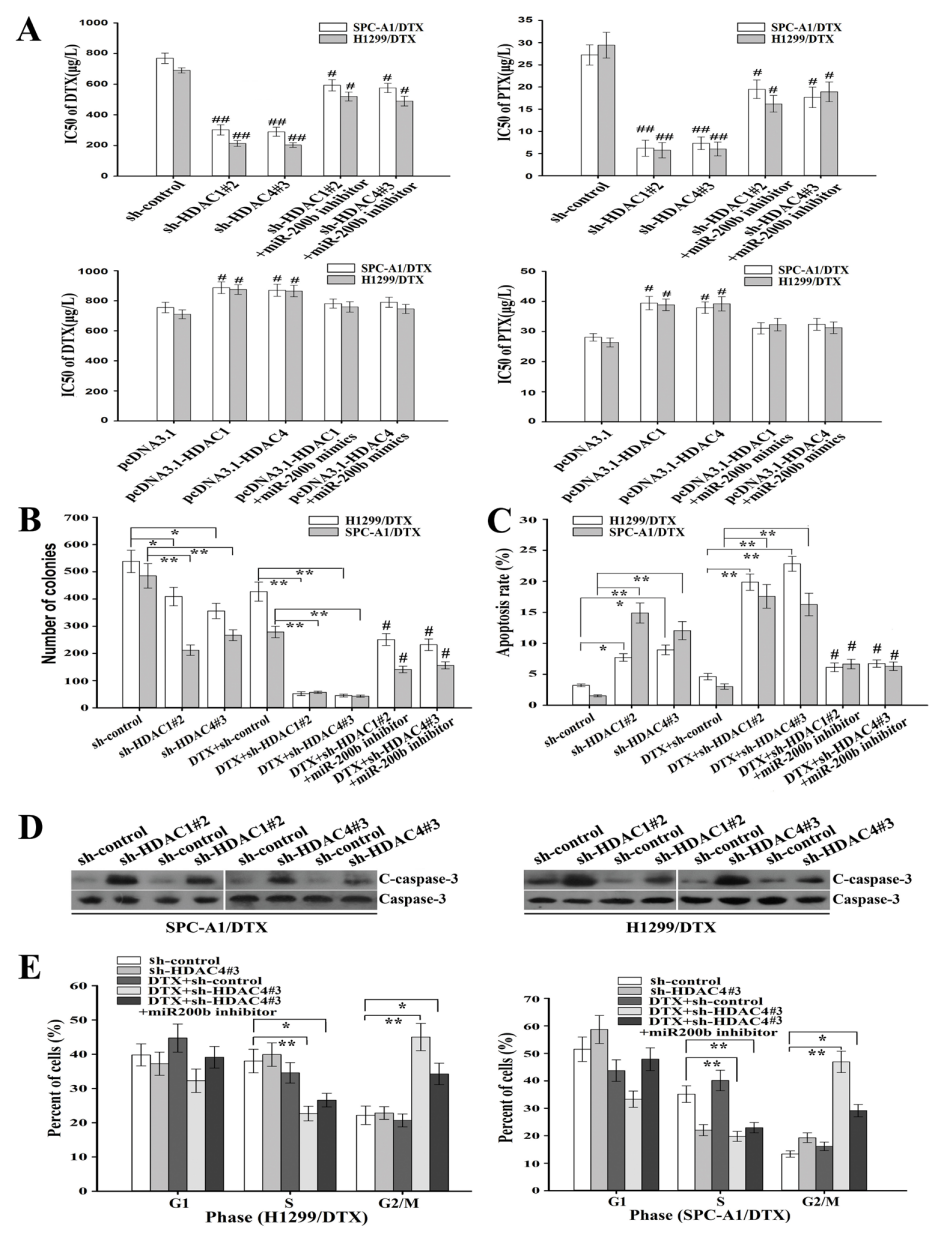
Inhibition of HDAC1/4 reverses chemoresistance of docetaxel-resistant LAD cells partially in a miR-200b-dependent manner (A) The IC50 values for docetaxel or paclitaxel as determined by MTT assay after transfection of sh-control, sh-HDAC1#2 or sh-HDAC4#3 vectors into docetaxel-resistant LAD cells without (or with) previous transfection with miR-200b inhibitor. Upregulation of HDAC1 and HDAC4 reversed the chemoresistance of docetaxel-resistant LAD cells. (B) The colony formation assay was performed as described in Methods. The number of colonies was counted and compared. (C) Flow cytometric analysis of apoptosis after transfection of sh-control, sh-HDAC1#2 or sh-HDAC4#3 vector into H1299/DTX or SPC-A1/DTX cells, without (or with) previous transfection of miR-200b inhibitor. (D) Protein levels of cleaved caspase-3 as measured by western blot after transfection of sh-control, sh-HDAC1 or sh-HDAC4 vectors into H1299/DTX or SPC-A1/DTX cells, without (or with) previous transfection of miR-200b inhibitor. Total caspase-3 was used as an internal control. (E) Flow cytometry analysis of cell cycle in H1299/DTX or SPC-A1/DTX cells transfection with sh-control, sh-HDAC4#3 or combined with DTX treatment or co-transfected with miR-200b inhibitor. The data were the means ± standard error of at least three independent experiments. *P < 0.05, **P < 0.01; ^#^*P* < 0.05 *vs* control group.

To confirm the effects of HDAC1/4 on regulating the chemoresistance of LAD cells, sh-control, sh-HDAC1#2 or shRNA-HDAC4#3 vectors were transfected into H1299/DTX and SPC-A1/DTX cells without (or with) previous transfection with miR-200b inhibitor, and the IC_50_ values of docetaxel or paclitaxel were measured by MTT assay (Figure 5A). The IC_50_ values of docetaxel (or paclitaxel) in SPC-A1/DTX/sh-HDAC1#2, H1299/DTX/sh-HDAC1#2, SPC-A1/DTX/sh-HDAC4#

3 or H1299/DTX/sh-HDAC4#3 cells were significantly decreased (*P* < 0.01) compared with those in control cells. Nevertheless, the inhibition effects could be partially abrogated by miR-200b inhibitor (Figure 5A). However, the IC_50_ values of docetaxel (or paclitaxel) in SPC-A1/DTX/pcDNA3.1-HDAC1, H1299/DTX/pcDNA3.1-HDAC1, SPC-A1/DTX/pcDNA3.1-HDAC4 or H1299/DTX/pcDNA3.1-HDAC4 cells were significantly increased (*P* < 0.05) compared with those in control cells. Intriguingly, the effects of upregulation of HDAC1 or HDAC4 on the IC_50_ values could be partially abrogated by miR-200b mimics (Figure 5A). These data clearly indicated that upregulation of HDAC1/4 could promote chemoresistance of LAD cells.

Also, downregulation of HDAC1/4 significantly reduced the colony formation capacities of H1299/DTX and SPC-A1/DTX cell lines, and these effects were also partially reversed by miR-200b inhibitor (*P* < 0.01, Figure 5B; Supplementary Figure 2A). Next, flow cytometric assay was performed to evaluate apoptosis and cell cycle. When the cells were treated with 100 µg/L docetaxel, the apoptosis rate of H1299/DTX/sh-HDAC1#2, SPC-A1/DTX/sh-HDAC1#2, H1299/DTX/sh-HDAC4#3 and SPC-A1/DTX/sh-HDAC4#3 cell lines was significantly increased by 3.31-, 4.84-, 3.95- and 4.41-fold, respectively (*P* < 0.01, Figure 5C; Supplementary Figure 1B) compared with control cells. Likewise, the apoptosis-promoting effect was weakened when miR-200b inhibitor was previously transfected (Figure 5C; Supplementary Figure 2B). Similar effects of HDAC1/4 on the expression of cleaved-caspase-3 protein level were observed (Figure 5D). Cell cycle analysis showed that downregulation of HDAC4 dramatically increased the percentage of cells in G2/M phase in H1299/DTX and SPC-A1/DTX cells, which could be partially reversed by miR-200b inhibitor (*P* < 0.01, Figure 5E, Supplementary Figure 2C). Therefore, suppression of HDAC1/4 could reverse the *in vitro* chemoresistance of LAD cells, at least partially in a miR-200b-dependent manner.

### HDAC1/4/Sp1/miR-200b signaling is responsible for aberrant expression of E2F3, survivin and Aurora-A in docetaxel-resistant LAD cells

**Figure 6:**
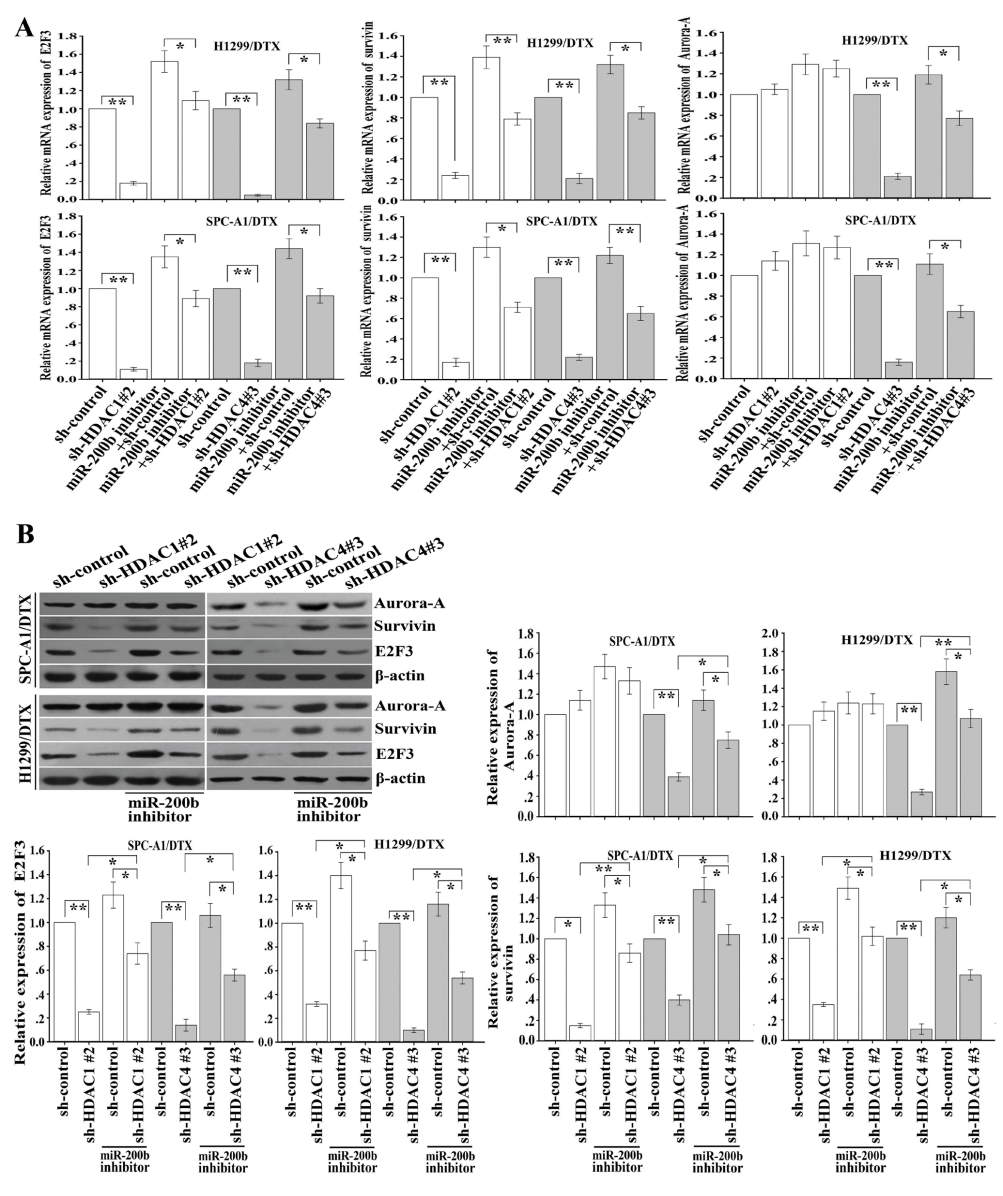
Downregulation of HDAC1/4 suppresses expression of E2F3, survivin and Aurora-A partially through the miR-200b-dependent pathway (A) Relative mRNA expression of E2F3, survivin and Aurora-A as measured by qRT-PCR after transfection of sh-control, sh-HDAC1#2 or sh-HDAC4#3 vectors into H1299/DTX and SPC-A1/DTX cells, without (or with) previous transfection of miR-200b inhibitor. Data were normalized by GAPDH and determined relative to sh-control vector group. (B) Protein levels of E2F3, survivin and Aurora-A as measured by western blot after transfection of shRNA-control, shRNA-HDAC1#2 or shRNA-HDAC4#3 vectors into H1299/DTX or SPC-A1/DTX cells, without (or with) previous transfection of miR-200b inhibitor. β-actin was used as an internal control. Data were the means ± standard error of at least three independent experiments. *P < 0.05, **P < 0.01.

E2F3, a functional target of miR-200b [18], plays crucial roles in regulating the chemoresistance of LAD cells. Next we focused on whether the HDAC1/4/Sp1/miR-200b pathway might be involved in the regulation of E2F3 and its downstream target genes. qRT-PCR and western blot assays indicated that inhibition of HDAC4 led to the decreased mRNA and protein levels of E2F3, Aurora-A and survivin (Figure 6A, 6B), and the downregulating effects could be partially rescued by miR-200b inhibitor (Figure 6A, 6B). Likewise, silencing of HDAC1 also led to the decreased expression of survivin mRNA and protein (Figure 6A, 6B). However, HDAC1 repression had no significant effect on Aurora-A in H1299/DTX and SPC-A1/DTX cells (Figure 6A, 6B).

**Figure 7:**
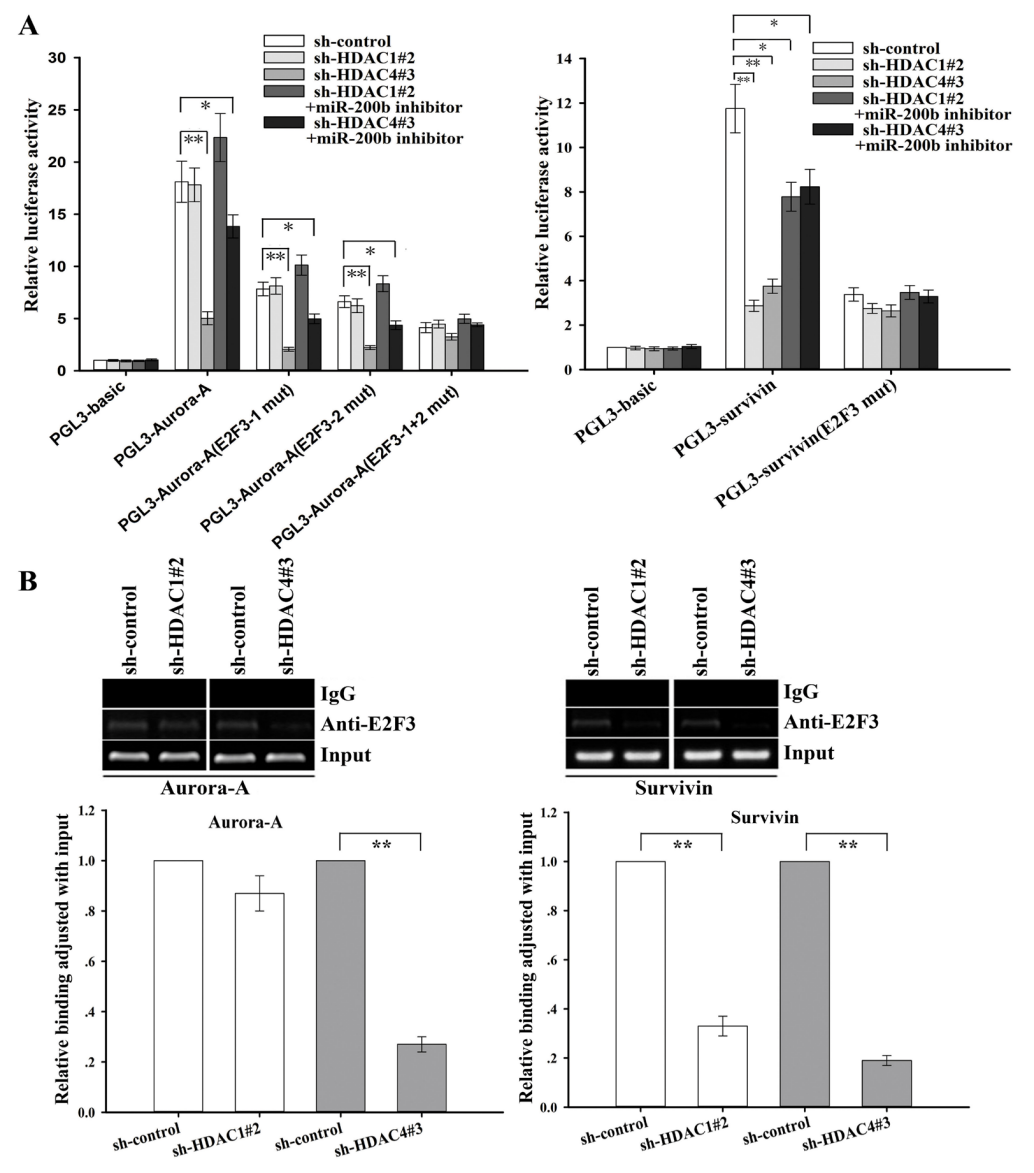
HDAC1/4 involvement in the regulation of survivin or Aurora-A through the miR-200b/E2F3-dependent pathway (SPC-A1/DTX cells) (A) Luciferase activity assays revealed that downregulation of HDAC1/4 significantly elevated Aurora-A (left) or survivin (right) promoter activity partially in a miR-200b/E2F3-dependent manner. Data were normalized by Renilla luciferase activity and determined relative to empty vector group. (B) Downregulation of HDAC1 and HDAC4 decreases the amount of E2F3 binding to the survivin promoter, while suppression of HDAC4 reduces the amount of E2F3 binding to the Aurora-A promoter. ChIP assays were performed with E2F3 antibody in SPC-A1/DTX cells transfected with sh-control and either sh-HDAC1#2 or sh-HDAC4#3. Immunoprecipitated DNA was amplified by qRT-PCR with primers designed to amplify the sequences containing the putative E2F3-binding sites. Data were normalized to qRT-PCR products amplified with input DNA before immunoprecipitation. Data were the means ± standard error of at least three independent experiments. *P < 0.05, **P < 0.01.

To further determine the roles of HDAC1/4/Sp1/miR-200b signaling in the regulation of E2F3-related target genes, we analyzed the genomic sequences of promoters of Aurora-A and survivin as reported in previous studies [29, 30], and found multiple binding sites for the E2F3 transcription factor in Aurora-A and survivin promoters. We cloned the fragments of the promoters containing E2F3-binding sites into the pGL3 basic firefly luciferase reporter, and luciferase assays showed that the promoters of Aurora-A and survivin were active in SPC-A1/DTX cells (Figure 7A). To confirm the effects of HDAC1/4 on the promoters of Aurora-A and survivin, sh-control, sh-HDAC1 or sh-HDAC4 vector was transfected into SPC-A1/DTX cells that were cotransfected with Renilla and either wild-type (Aurora-A or survivin), E2F3 binding mutant or empty vector firefly promoter reporter constructs. HDAC4 suppression significantly decreased the luciferase activity of the Aurora-A and survivin promoters (*P* < 0.01, Figure 7A), and the downregulating effect was partially attenuated by miR-200b inhibitor. A similar effect of HDAC1 inhibition on regulation of survivin, but not Aurora-A, could be also observed in SPC-A1/DTX cells (Figure 7A). However, the effects of HDAC1/4 inhibition on survivin or Aurora-A promoters were almost abolished in constructs in which the E2F3 binding sites were mutated (Figure 7A). Together these data indicate that HDAC1/4/Sp1/miR-200b signaling regulated the promoter activity of Aurora-A and survivin in an E2F3-dependent manner.

Next, ChIP assay was performed to determine the effect of HDAC1/4 on the *in vivo* ability of E2F3 binding to the promoter of Aurora-A or survivin. As shown in Figure 7B, inhibition of HDAC4 but not HDAC1 significantly reduced the amount of E2F3 binding to the promoter of Aurora-A, while downregulation of both of HDAC4 and HDAC1 significantly reduced the amount of E2F3 binding to the promoter of survivin. These data suggest that the HDAC1/4/Sp1/miR-200b signaling plays a critical role in the regulation of E2F3, survivin and Aurora-A in docetaxel-resistant LAD cells.

### Downregulation of HDAC1/4 or upregulation of miR-200b chemosensitizes docetaxel-resistant LAD cells in vivo

**Figure 8:**
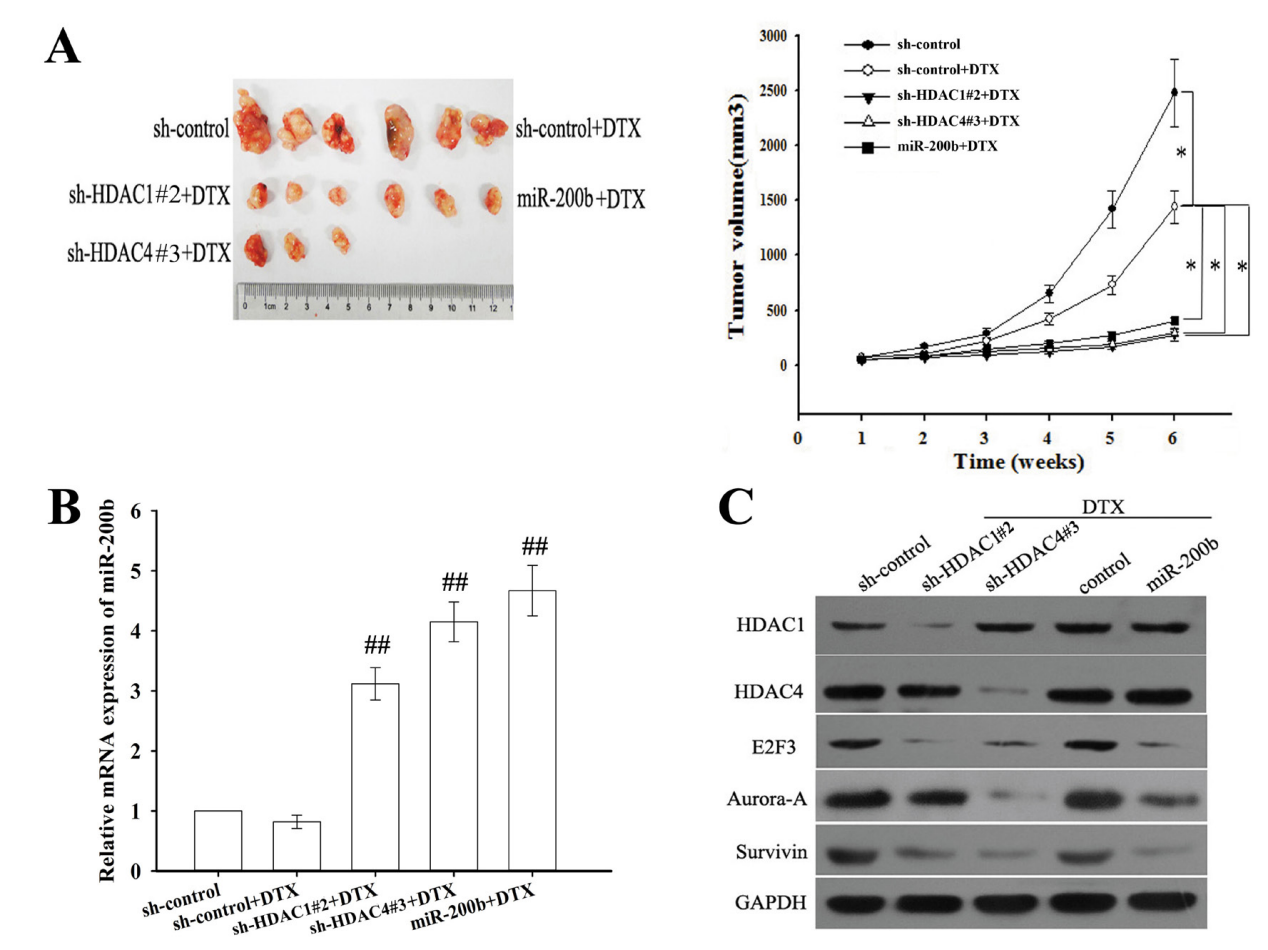
Suppression of HDAC1/4 or upregulation of miR-200b reverses in vivo chemoresistance of docetaxel-resistant LAD cells (H1299/DTX cells) (A) Growth of tumors in nude mice subcutaneously transplanted with H1299/DTX cells stably transfected with sh-control, sh-HDAC1#2, sh-HDAC4#3 or miR-200b vectors (six mice in each group). Representative photographs of tumors were obtained 6 weeks after inoculation. Data were the means ± standard error. *P < 0.05. (B) The relative mRNA level of miR-200b as measured by qRT-PCR in tumors 6 weeks after inoculation. Data were normalized to U6 RNA and determined relative to control group Data were the means ± standard error. ^##^P < 0.01 vs. control group. (C) HDAC1, HDAC4, E2F3, survivin and Aurora-A protein levels as measured by western blot in tumors 6 weeks after inoculation. GAPDH was used as an internal control.

To further confirm the effect of HDAC1 or HDAC4 on the *in vivo* chemosensitivity of LAD cells, H1299/DTX cells stably transfected with various constructs were subcutaneously transplanted into nude mice. When treated with docetaxel, tumors derived from H1299/DTX cells stably transfected with sh-HDAC1#2, sh-HDAC4#3 or miR-200b vector grew more slowly than controls (Figure 8A). The expression level of miR-200b was upregulated in sh-HDAC1#2, sh-HDAC4#3 and miR-200b vector groups compared with the control groups (Figure 8B). As shown in Figure 8C and Supplementary Figure 3, the expression levels of HDAC1 and HDAC4 were significantly downregulated in sh-HDAC1#2 and sh-HDAC4#3 groups. Furthermore, E2F3 and survivin levels were downregulated in sh-HDAC1#2, sh-HDAC4#3 and miR-200b vector groups, but Aurora-A level was only downregulated in sh-HDAC4#3 and miR-200b vector groups (Figure 8C and Supplementary Figure 3).

Immunostaining showed that the positive rates of PCNA and Ki67 in sh-HDAC1#2, sh-HDAC4#3 and miR-200b vector groups were significantly decreased compared with those in the control group (Supplementary Figure 3). TUNEL staining revealed increased apoptotic cells in tumors generated from sh-HDAC1#2, sh-HDAC4#3 and miR-200b vector groups compared with the control group (Supplementary Figure 3). These results indicate that suppression of HDAC1/4 or upregulation of miR-200b chemosensitizes docetaxel-resistant LAD cells *in vivo*.

### HDAC1/4, inversely correlated with miR-200b, is upregulated in the docetaxel-insensitive LAD tissues compared with the docetaxel-sensitive LAD tissues

**Figure 9:**
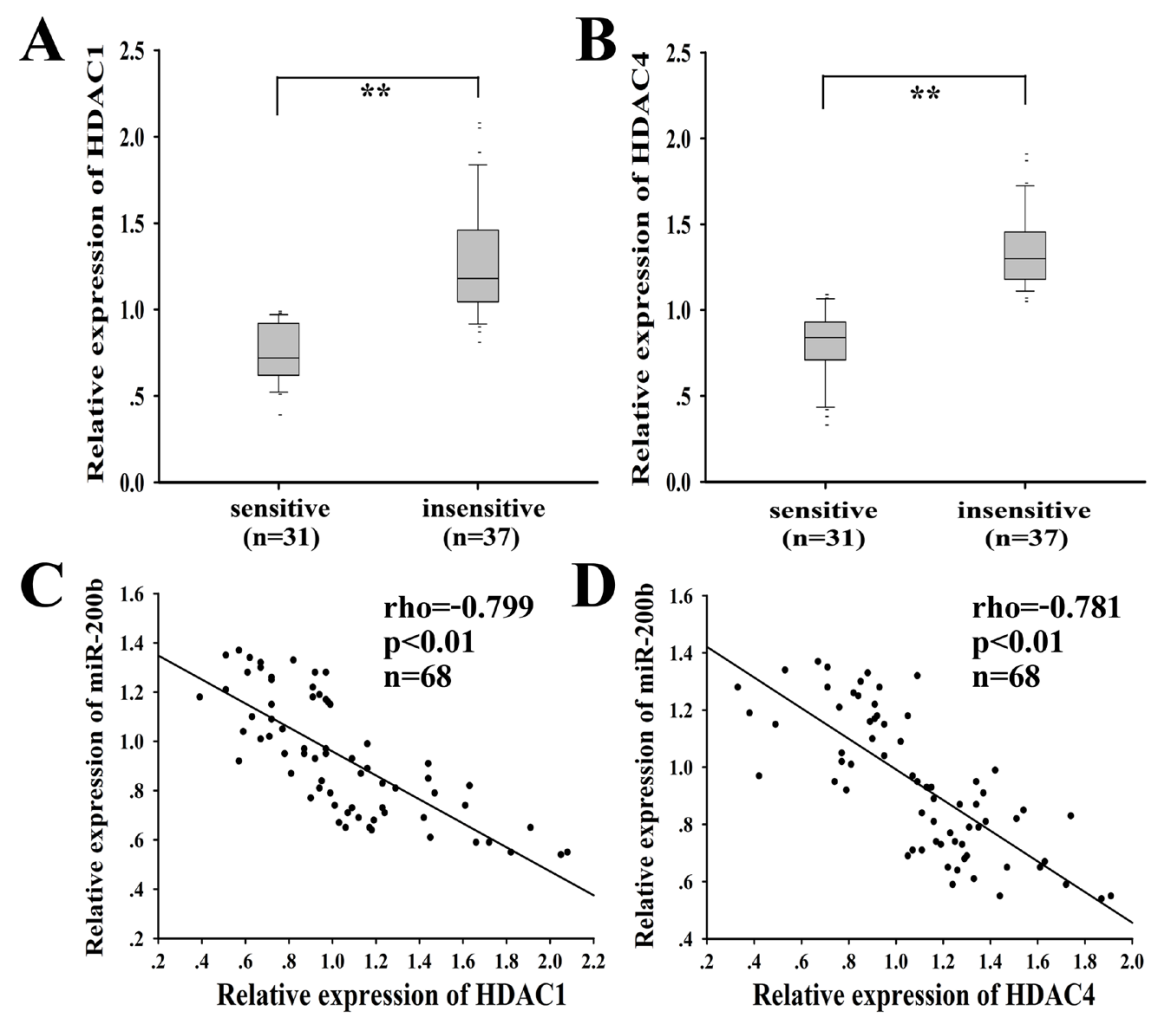
HDAC1/4, inversely correlated with miR-200b, is upregulated in docetaxel-insensitive human LAD cases compared with docetaxel-sensitive LAD cases qRT-PCR was performed to detect the relative mRNA level of HDAC1 (A) and HDAC4 (B) in docetaxel-sensitive (n=31) and insensitive (n=37) LAD tissues. HDAC1 (C) or HDAC4 (D) and miR-200b mRNA levels were inversely correlated in 68 LAD tissues as determined by linear regression analysis. Spearman rank test rho and *P* values (2-tailed) were shown. The HDAC1/4 level was normalized to GAPDH and miR-200b was normalized to U6. **P < 0.01.

A total of 68 cases of clinical LAD tissues were obtained from patients at advanced stage and divided into “sensitive” (complete or partial response, CR+PR) and “insensitive” (stable or progressive disease, SD+PD) groups based on the response to the docetaxel-based chemotherapies. The clinicopathological factors of LAD patients are shown in Supplementary Table 5. HDAC1/4 was significantly upregulated in the docetaxel-insensitive group (n=37) compared with the docetaxel-sensitive group (n=31) (Fig. 9A and 9B). ROC curve analysis was performed to establish the optimal cutoff values for the HSCORE of HDAC1 and HDAC4 expression level, which yielded a value of 102.3 and 78.3, respectively (data not shown). Statistical analysis showed that high HDAC1 or HDAC4 mRNA expression was significantly correlated with advanced clinical stage (*P*=0.003 or 0.030, respectively) and poor tumor response of patients (*P*=0.004 or 0.001, respectively). A statistically significant inverse correlation was observed between HDAC1 and miR-200b (r=-0.799, *P* < 0.01; Fig. 9C). A statistically significant inverse correlation was also observed between HDAC4 and miR-200b (r=-0.781, *P* < 0.01; Fig. 9D).

In addition, the correlations of HDAC1/4 expression with survival of patients were statistically analyzed. Patients with a high HDAC1 expression (HSCORE≥102.3) had a significantly shorter progression-free survival (PFS) than did those with a low HDAC1 expression (HSCORE<102.3) (*P*=0.0185; Supplementary Figure 4A). Likewise, patients with a high level of HDAC4 expression (HSCORE≥78.3) had a significantly shorter PFS than did those with a low level of HDAC4 expression (HSCORE<78.3) (*P*=0.0032; Supplementary Figure 4B). Thus, upregulation of HDAC1/4, inversely correlated with downregulation of miR-200b in LAD tissues, was correlated with poor survival of patients who received docetaxel-based chemotherapies.

## DISCUSSION

In the current study, we present several novel findings: (1) HDAC inhibitors restore the expression of miR-200b and reverse chemoresistance of docetaxel-resistant LAD cells, (2) HDAC1/4 levels are upregulated in docetaxel-resistant LAD cells compared with parental cells, and inhibition of HDAC1/4 significantly increases the level of miR-200b and upregulates histone H3-acetylation level at miR-200b promoters partially via a Sp1-dependent pathway, (3) negative regulation of HDAC1/4 suppresses cell proliferation, promotes cell apoptosis, induces G2/M cell cycle arrest and ultimately reverses *in vitro* chemoresistance of docetaxel-resistant LAD cells partially in a miR-200b-dependent manner, (4) HDAC1/4 suppression-induced miR-200b expression contributes to the downregulation of E2F3, survivin and Aurora-A, and upregulation of cleaved-caspase-3, (5) downregulation of HDAC1/4 or upregulation of miR-200b reverses *in vivo* chemoresistance of docetaxel-resistant LAD cells, and (6) HDAC1/4, inversely correlated with miR-200b, is upregulated in docetaxel-insensitive LAD tissues compared with docetaxel-sensitive LAD tissues. Taken together, we conclude that the novel HDAC1/4/Sp1/miR-200b/E2F3 regulatory axis contributes to chemoresistance of docetaxel-resistant LAD cells.

Aberrant histone hypoacetylation at the promoters of numerous tumor suppressors has been reported in previous studies [26, 31]. Based on the homology to yeast proteins, the family of HDAC enzymes is classified into three main classes: class I (HDAC1, 2, 3 and 8), class II (HDAC4, 5, 7, 9, 6 and 10) and class IV (HDAC 11) [32]. So far, inhibitors of class I, II and IV HDACs have been intensively researched, and two HDAC inhibitors, vorinostat and romidepsin, have already been approved by the US Food and Drug Administration for cancer treatment [33]. The two HDAC inhibitors, VPA, quite active against HDACs 1–5, 7 and 9, and TSA, efficient against HDACs 1–7 and 9 [33], were used in the present study. Both TSA and VPA significantly restored the expression of miR-200b and reversed chemoresistance of docetaxel-resistant LAD cells. Generally, HDACs lack a DNA binding domain and are unable to bind to DNA directly, and thus they interact with DNA through specific proteins.

Sp1 is a highly evolutionarily conserved transcription factor that specifically recognizes the GC box (GGGGCGGGG). Sp1 participates in the transcriptional regulation of genes associated with cell proliferation, apoptosis, differentiation, and transformation [34-36]. Sp1 can recruit HDAC4 to the promoter of miR-200a, resulting in silencing of miR-200a and promoting the proliferation and migration of hepatocellular carcinoma cells [28]. Also, Sp1 is able to recruit HDAC1 to the promoter of phosphatase and tension homolog deleted on chromosome ten (PTEN), which then inhibits PTEN expression and enhances cancer cell migration and invasion [37]. In this study, we identified multiple Sp1 binding sites in the two promoters of miR-200b, and inhibition of HDAC1 and HDAC4 significantly increased luciferase activities of luciferase constructs with the two indicated promoters in a Sp1-dependent manner. Furthermore, we found that Sp1 was able to recruit HDAC1/4 to the promoters of miR-200b *in vivo*, and inhibition of HDAC1/4 increased the histone-H3 acetylation level at the miR-200b promoters in a Sp1-dependent manner. Here, p21 was used as a positive control [38]. Our study also revealed that HDAC1 and HDAC4 are significantly upregulated in docetaxel-resistant LAD cells compared with parental cells, and inhibition of HDAC1 or HDAC4 significantly enhances miR-200b levels, at least partially in a Sp1-dependent manner. Thus, these results suggest that Sp1 is a crucial transcription factor responsible for regulating miR-200b expression by HDAC1/4 in docetaxel-resistant LAD cells.

E2F3 transcription factor, a member of the E2F family, has been reported to play an important role in the regulation of cell proliferation, clonogenic activity, survival and cell cycle distribution [39-42]. Our previous work demonstrated that suppression of E2F3 is a potential mechanism by which miR-200b reverses chemoresistance of docetaxel-resistant LAD cells [18]. In the present work, we showed that downregulation of HDAC1/4 inhibited E2F3 expression, suppressed cell proliferation, promoted cell apoptosis, and induced G2/M cell cycle arrest partially through the miR-200b pathway. Generally, HDAC repression activates gene transcription through forming an open chromatin structure. However, HDAC inhibition can also induce repressive histone modifications through recruitment of a corepressor complex such as pRB to the promoter region [43]. Other reports have shown that an antitumor dose of HDAC inhibitors can only affect less than 10% of the expressed genes in malignant cells [44]. Here, HDAC1 repression significantly decreased E2F3 expression, which might be largely because of the following: first, HDAC1 might reduce E2F3 expression by recruitment of the corepressor complex to the E2F3 promoter region; second, HDAC1 might not be the specific HDAC responsible for histone modifications at the promoter region of E2F3, and instead HDAC1 decreases E2F3 expression by upregulation of certain miRNAs including miR-200b, which then post-transcriptionally regulate the expression of E2F3 gene.

Aurora-A, a Ser/Thr kinase involved in regulating cell cycle progression, and survivin, an apoptosis-associated gene, have been reported to be transcriptionally activated by the E2F3 transcription factor [29, 30, 45]. In our study, we showed that both HDAC1 and HDAC4 repression significantly inhibited survivin expression by reducing the activity of the survivin promoter via downregulating E2F3 expression in a miR-200b-dependent manner and reducing the amount of E2F3 protein binding to this promoter. Interestingly, only HDAC4 but not HDAC1 repression could inhibit Aurora-A expression by reducing the activity of Aurora-A promoter via downregulating E2F3 expression in a miR-200b-dependent manner and thus reducing the amount of E2F3 protein binding to this promoter. Possible reasons for the inability of HDAC1, an upstream regulator of E2F3, to decrease Aurora-A in docetaxel-resistant LAD cells are as follows. First, a growing body of literature indicates that long non-coding RNAs (lncRNAs) are key players in gene regulation, therefore lncRNAs and HDAC1 may act in a coordinated fashion to regulate gene expression, which may offset the effects of E2F3 on the regulation of Aurora-A. Second, except for E2F3, HDAC1 and other transcription factors such as E4TF1, hypoxia-inducible factor-1α and p53 may directly or indirectly act in a coordinated fashion to regulate Aurora-A expression. The precise reason underlying the lack of HDAC1 effect on Aurora-A expression is still unclear and needs to be elucidated in future research. Together these findings suggest that repression of HDAC1/4 suppresses cell proliferation, promotes cell apoptosis, and induces G2/M cell cycle arrest by downregulation of E2F3, Aurora-A and survivin, at least partially through the miR-200b-dependent pathway.

To further illustrate the roles of HDAC1/4/miR-200 in docetaxel-resistance of LAD patients, we evaluated the expression of HDAC1/4 in LAD tissues from patients who received docetaxel-based chemotherapies, and showed that the expression level of HDAC1/4 mRNA in docetaxel-sensitive LAD tissues was significantly lower than that in docetaxel-insensitive tissues. Meanwhile, HDAC1/4 was inversely correlated with miR-200b in LAD tissues. We further showed that high HDAC1/4 expression was correlated with poor PFS of LAD patients. Taken together, these data, along with previous observations that downregulation of miR-200b was involved in docetaxel resistance of LAD, support the hypothesis that the novel HDAC1/4/miR-200b/E2F3 signaling contributes to chemoresistance of LAD cells. Therefore, targeting this signaling is a potential strategy for reversing the chemoresistance in human LAD.

## MATERIALS AND METHODS

### Ethics statement

Investigation has been conducted in accordance with the ethical standards and according to the Declaration of Helsinki and according to national and international guidelines and has been approved by the authors' institutional review board.

### Cell lines and animals

Docetaxel-resistant SPC-A1 cells (SPC-A1/DTX) were established as described in our previous work [46] and preserved in 50.0 μg/L docetaxel. Docetaxel-resistant H1299 cells (H1299/DTX) were established and preserved in 50.0 μg/L docetaxel, and grown as previously described [47]. BALB/c athymic nude mice (male, SPF, 4-6 weeks) were provided by the Department of Comparative Medicine in Jinling Hospital.

### Clinical tissue samples

A total of 68 LAD tissues were obtained from patients with LAD at advanced stage in our hospital between January 2006 and June 2007. All patients met all of the following criteria: histological diagnosis of primary LAD with at least one measurable lesion; clinical stage IIIB–IV; and first-line chemotherapy either with docetaxel 75 mg/m^2^ and cisplatin 100 mg/m^2^ or docetaxel 75 mg/m^2^ and carboplatin area under the curve 6 mg/mL/min administered every 3 weeks for a maximum of 5 cycles [18]. Tissue samples were divided into “sensitive” (complete or partial response) and “insensitive” (stable or progressive disease) groups based on the patient responses assessed by medical image analysis and detection of serum tumor markers after 4 or 5 cycles of docetaxel-based chemotherapy [18].

### Construction of plasmids and transfection of oligonucleotides or plasmids

The pcDNA3.1-HDAC1 and pcDNA3.1-HDAC4 plasmids were kindly provided by Prof. Jun Lu (Northeast Normal University, China) and Professor Shu-han Sun (Second Military Medical University, China) [28], respectively. The two promoters of miR-200b, located ~4 kb and ~2 kb upstream of the 5′-stem loop, have been reported in previous studies [20, 48]. The core regions of the two promoters are located at chr1:1087797-1088137 (341 bp) and chr1:1089316-1090354 (1039 bp (UCSC Genome Browser, March 2006) [20, 48]. The two core regions were amplified by PCR and cloned into the pGL3-basic vector (Promega, San Luis, CA, USA) using KpnI and HindIII sites, and the resulting plasmids were named (pGL3) promoter-1 and promoter-2. Two open databases (Consite and PROMO) were used to analyze the core regions of the two promoters, and multiple Sp1 binding sites were detected Mutation of Sp1 bingding site-mutant constructs were generated by use of the QuikChange XL site-directed mutagenesis kit from Stratagene (La Jolla, CA, USA). All primer sequences are presented in Supplementary Table 1. All vectors were confirmed by DNA sequencing.

The single core region of the Aurora-A promoter (–96/+354), as confirmed in a previous work [29], was cloned into the pGL3-basic vector using MluI and BglII sites. This core region contains E2F3 binding sites [29]. The primer pairs used for generating the E2F3 binding site mutant pGL3-Aurora-A (–96/+354) vectors (E2F3-1mut and E2F3-2mut) are presented in Supplementary Table 1. The DNA fragment corresponding to –165/+1 of the survivin promoter amplified by PCR was cloned into the pGL3-basic vector using KpnI and HindIII sites. The primer pairs are presented in Supplementary Table 1. All vectors were confirmed by DNA sequencing.

The siRNA-HDACs, siRNA-NC, miR-200b inhibitor, miR-200b inhibitor control, miR-200b mimics, miR-200b mimics control and siRNA-Sp1 were purchased from GenePharma (Shanghai, China). The primers pairs used for sh-control, sh-HDAC1 and sh-HDAC4 are presented in Supplementary Table 2. All sh-RNA vectors were confirmed by DNA sequencing. Cells were transfected with si-RNA Mate (GenePharma, China) or Turbofect Transfection Reagent (Thermo Scientific, USA), according to the manufacturer's instructions.

### Real-time quantitative reverse-transcription polymerase chain reaction (qRT-PCR)

Total RNA was extracted with TRIzol reagent (Takara, Japan) and concentration was measured by a spectrophotometer. Reverse transcription was conducted using the PrimeScript RT Reagent Kit (Takara) according to the manufacturer's instructions. Quantitative real-time PCR was performed with the SYBR PrimeScript RT-PCR Kit (Takara) based on the manufacturer's instructions. The miR-200b or mRNA levels normalized to U6 rRNA or GAPDH mRNA, respectively, were calculated with the 2^-ΔΔCt^ method. The expression levels were relative to the fold change of the corresponding control cells defined as 1.0. All primer pairs are presented in Supplementary Table 3.

### Drug sensitivity assay

Drug sensitivity was measured by 3-(4,5-dimethylthiazol-2-yl)-2,5-diphenyl-tetrazolium bromide (MTT, Sigma, USA) assay as previously described [18].

### Colony formation assay

Colony formation assay was conducted as previously described [47].

### Western blot

Total protein lysate was separated by sodium dodecyl sulfate-polyacrylamide gel electrophoresis (SDS-PAGE) and transferred onto polyvinylidene fluoride (PVDF) membranes (Millipore, USA). Membranes were blocked with 5% skim milk in TBST for 2 h at room temperature and incubated with primary antibodies overnight at 4°C. Membranes were washed with TBST (three washes, 5 min) and incubated with secondary antibodies at room temperature for 2 h. Membranes were then washed by TBST (four times, 10 min), and visualized with a chemiluminescence kit (Thermo Scientific). Antibodies against HDAC2 (1.0 µg/ml, polyclonal rabbit IgG, immunogen affinity purified), HDAC3 (1.0 µg/ml, polyclonal rabbit IgG, immunogen affinity purified), HDAC5 (4.0 µg/ml, polyclonal rabbit IgG, immunogen affinity purified), HDAC6 (2.0 µg/ml, polyclonal rabbit IgG, immunogen affinity purified), HDAC7 (0.5 µg/ml, polyclonal rabbit IgG, immunogen affinity purified), HDAC8 (1:1000, polyclonal rabbit IgG, immunogen affinity purified), HDAC9 (2.0 µg/ml, polyclonal rabbit IgG, immunogen affinity purified), HDAC10 (1:1000, polyclonal rabbit IgG, immunogen affinity purified), HDAC11 (2.0 µg/ml, polyclonal rabbit IgG, immunogen affinity purified), survivin (2.0 µg/ml, polyclonal rabbit IgG, protein A purified), cleaved caspase-3 (2.0 µg/ml, polyclonal rabbit IgG, immunogen affinity purified), acetyl-histone H3 (1:500, polyclonal rabbit IgG, immunogen affinity purified) were purchased from Abcam, Hong Kong. Antibodies against histone H3, HDAC1 (1:1000, monoclonal mouse IgG, immunogen affinity purified), HDAC4 (1:2000, monoclonal rabbit IgG, immunogen affinity purified), Sp1 (1:1000, polyclonal rabbit IgG, protein A purified), and Aurora-A (1:1000, monoclonal rabbit IgG, immunogen affinity purified) were purchased from Cell Signaling Technologies, USA. Antibodies against E2F3 (1:1000, monoclonal mouse IgG), β-actin (1:2000, monoclonal mouse IgG) and GAPDH (1:5000, monoclonal mouse IgG) were obtained from Santa Cruz Biotechnology, USA.

### Co-immunoprecipitation assay

Co-immunoprecipitation assay was performed with a Co-Immunoprecipitation (Co-IP) Kit (Thermo Fisher Scientific Inc, Rockford, USA) according to the manufacturer's instructions. Briefly, 75 µg of affinity-purified antibodies [Sp^1^ (polyclonal rabbit IgG, protein A purified), HDAC^1^ (monoclonal mouse IgG, immunogen affinity purified) and HDAC^4^ (monoclonal rabbit IgG, immunogen affinity purified), Cell Signaling Technology)] were coupled into the spin columns. Cell lysate was carefully prepared and pre-cleared using the control agarose resin. Next, the cell lysate was co-immunoprecipitated and eluted. Finally, samples were evaluated by western blot analysis.

### Chromatin Immunoprecipitation (ChIP) Assay

ChIP assay was performed with Immunoprecipitation Assay Kits (Millipore) according to the manufacturer's instructions. Briefly, cells were cross-linked with 1% formaldehyde for 10 min at 37°C. The cells were then resuspended in 200 μl of lysis buffer and incubated for 10 min on ice. The lysate was sheared to lengths between 200 and 1000 bp by sonication. The supernatant was pre-cleared with a Salmon Sperm DNA/Protein A Agarose-50% Slurry. The recovered supernatant was incubated with antibodies [Sp^1^ (1:^10^, polyclonal rabbit IgG, immunogen affinity purified), HDAC^1^ (1:^10^, monoclonal mouse IgG, protein G purified) and HDAC^4^ (15 µg, polyclonal rabbit IgG, immunogen affinity purified) (Abcam); acetyl-histone H^3^ (10 µg, rabbit polyclonal IgG, protein A purified), E[Bibr R2][Bibr R3] (2 µg, mouse monoclonal IgG, protein A purified) (Millipore)] or an isotype control IgG (15 µg rabbit polyclonal or 5 µg mouse monoclonal IgG, protein A purified) (Millipore) overnight at 4°C with rotation. The antibody/DNA complex was collected using Salmon Sperm DNA/Protein A Agarose Slurry for 1 h at 4°C with rotation, and the complex was eluted by elution buffer. Crosslinks were reversed with 5M NaCl heating at 65°C for 4 h. The DNA sample was then purified and measured by qRT-PCR. The primers are listed in Supplementary Table 4.

### Dual luciferase reporter assay

SPC-A1/DTX or H1299/DTX cells (4×10^3^/well) were seeded into 96-well plates and cotransfected with luciferase reporter plasmids and sh-RNAs with (or without) miR-200b inhibitors. The Renilla-TK plasmid (Promega) was cotransfected into all samples. After 48 h, luciferase activities were measured with the Dual-Luciferase Reporter Assay kit (Promega). The relative luciferase activities were normalized by Renilla luciferase activities. The data were relative to the fold change of the corresponding control groups defined as 1.0.

### Flow cytometric analysis of apoptosis and cell cycle

Flow cytometric analyses of apoptosis and cell cycle were performed with Annexin V: FITC Apoptosis Detection Kits and PI/RNase staining buffer (BD Biosciences, USA), respectively, according to the manufacturer's instructions.

### Xenograft transplantation and immunostaining

Approximately 5.0×10^6^ H1299/DTX cells stably transfected with sh-control, sh-HDAC1, sh-HDAC4 or miR-200b vectors were subcutaneously transplanted into the right side of the posterior flank of nude mice. Tumor volumes were measured as previously described [18]. When the tumor size reached about 50 mm^3^, 1.0 mg/kg docetaxel, one dose every other day, with three doses in total, was administered by intraperitoneal injection [18]. After 6 weeks, all mice were sacrificed, and tumor tissues were used for the subsequent studies. Hematoxylin and eosin (H&;E) staining, immunostaining of Ki67 (1:500, monoclonal rabbit IgG, tissue culture supernatant), proliferating cell nuclear antigen (PCNA) (1 µg/ml, monoclonal mouse IgG, IgG fraction), HDAC1 (5 µg/ml, monoclonal mouse IgG, protein G purified), HDAC4 (5 µg/ml, polyclonal rabbit IgG, immunogen affinity purified), E2F3 (5 µg/ml, polyclonal rabbit IgG, immunogen affinity purified), Aurora-A (1:500, polyclonal rabbit IgG, immunogen affinity purified) and survivin (1:1000, polyclonal rabbit IgG, protein A purified) (Abcam) staining and TUNEL staining were performed according to the manufacturer's instructions.

### Statistical analysis

Data are shown as the means ± standard error of at least three independent experiments. The SPSS 17.0 software (SPSS Inc., Chicago, IL, USA) was used for statistical analysis. Multiple group comparisons were analyzed with one-way ANOVA and two group comparisons were performed with a Student *t* test. All tests performed were two-sided. Progression-free survival (PFS) was assessed from the first day of chemotherapy administration to the date of objective disease progression. The probability of survival was plotted by the Kaplan-Meier method and compared by the log-rank test. *P* < 0.05 was considered statistically significant.

## SUPPLEMENTARY FIGURES AND TABLE





## References

[1] Society AC (2013). Cancer Facts &; Figures 2013. Atlanta: American Cancer Society.

[R2] Siegel R, Naishadham D, Jemal A (2013). Cancer statistics, 2013. CA Cancer J Clin.

[R3] Chen Y, Jacamo R, Konopleva M, Garzon R, Croce C, Andreeff M (2013). CXCR4 downregulation of let-7a drives chemoresistance in acute myeloid leukemia. J Clin Invest.

[4] Rao E, Jiang C, Ji M, Huang X, Iqbal J, Lenz G, Wright G, Staudt LM, Zhao Y, McKeithan TW, Chan WC, Fu K (2012). The miRNA-17 approximately 92 cluster mediates chemoresistance and enhances tumor growth in mantle cell lymphoma via PI3K/AKT pathway activation. Leukemia.

[5] Siemens H, Jackstadt R, Kaller M, Hermeking H (2013). Repression of c-Kit by p53 is mediated by miR-34 and is associated with reduced chemoresistance, migration and stemness. Oncotarget.

[6] Ory B, Ellisen LW (2011). A microRNA-dependent circuit controlling p63/p73 homeostasis: p53 family cross-talk meets therapeutic opportunity. Oncotarget.

[7] Bier A, Giladi N, Kronfeld N, Lee HK, Cazacu S, Finniss S, Xiang C, Poisson L, de Carvalho AC, Slavin S, Jacoby E, Yalon M, Toren A, Mikkelsen T, Brodie C (2013). MicroRNA-137 is downregulated in glioblastoma and inhibits the stemness of glioma stem cells by targeting RTVP-1. Oncotarget.

[8] Ory B, Ramsey MR, Wilson C, Vadysirisack DD, Forster N, Rocco JW, Rothenberg SM, Ellisen LW (2011). A microRNA-dependent program controls p53-independent survival and chemosensitivity in human and murine squamous cell carcinoma. J Clin Invest.

[9] Wang Q, Chen W, Bai L, Chen W, Padilla MT, Lin AS, Shi S, Wang X, Lin Y (2014). Receptor-interacting protein 1 increases chemoresistance by maintaining inhibitor of apoptosis protein levels and reducing reactive oxygen species through a microRNA-146a-mediated catalase pathway. J Biol Chem.

[10] Zhu X, Li Y, Xie C, Yin X, Liu Y, Cao Y, Fang Y, Lin X, Xu Y, Xu W, Shen H, Wen J (2014 Feb 8). miR-145 sensitizes ovarian cancer cells to paclitaxel by targeting Sp1 and Cdk6. Int J Cancer.

[11] Feng X, Wang Z, Fillmore R, Xi Y (2014). MiR-200, a new star miRNA in human cancer. Cancer Lett.

[12] Bitarte N, Bandres E, Boni V, Zarate R, Rodriguez J, Gonzalez-Huarriz M, Lopez I, Javier Sola J, Alonso MM, Fortes P, Garcia-Foncillas J (2011). MicroRNA-451 is involved in the self-renewal, tumorigenicity, and chemoresistance of colorectal cancer stem cells. Stem Cells.

[13] Iliopoulos D, Lindahl-Allen M, Polytarchou C, Hirsch HA, Tsichlis PN, Struhl K (2010). Loss of miR-200 inhibition of Suz12 leads to polycomb-mediated repression required for the formation and maintenance of cancer stem cells. Mol Cell.

[14] Uhlmann S, Zhang JD, Schwager A, Mannsperger H, Riazalhosseini Y, Burmester S, Ward A, Korf U, Wiemann S, Sahin O (2010). miR-200bc/429 cluster targets PLCgamma1 and differentially regulates proliferation and EGF-driven invasion than miR-200a/141 in breast cancer. Oncogene.

[15] Li XL, Hara T, Choi Y, Subramanian M, Francis P, Bilke S, Walker RL, Pineda M, Zhu Y, Yang Y, Luo J, Wakefield LM, Brabletz T, Park BH, Sharma S, Chowdhury D (2014). A p21-ZEB1 complex inhibits epithelial-mesenchymal transition through the microRNA 183-96-182 cluster. Mol Cell Biol.

[16] Liu YN, Yin JJ, Abou-Kheir W, Hynes PG, Casey OM, Fang L, Yi M, Stephens RM, Seng V, Sheppard-Tillman H, Martin P, Kelly K (2013). MiR-1 and miR-200 inhibit EMT via Slug-dependent and tumorigenesis via Slug-independent mechanisms. Oncogene.

[17] Sun L, Yao Y, Liu B, Lin Z, Lin L, Yang M, Zhang W, Chen W, Pan C, Liu Q, Song E, Li J (2012). MiR-200b and miR-15b regulate chemotherapy-induced epithelial-mesenchymal transition in human tongue cancer cells by targeting BMI1. Oncogene.

[18] Feng B, Wang R, Song HZ, Chen LB (2012). MicroRNA-200b reverses chemoresistance of docetaxel-resistant human lung adenocarcinoma cells by targeting E2F3. Cancer.

[19] Tellez CS, Juri DE, Do K, Bernauer AM, Thomas CL, Damiani LA, Tessema M, Leng S, Belinsky SA (2011). EMT and stem cell-like properties associated with miR-205 and miR-200 epigenetic silencing are early manifestations during carcinogen-induced transformation of human lung epithelial cells. Cancer Res.

[20] Wee EJ, Peters K, Nair SS, Hulf T, Stein S, Wagner S, Bailey P, Lee SY, Qu WJ, Brewster B, French JD, Dobrovic A, Francis GD, Clark SJ, Brown MA (2012). Mapping the regulatory sequences controlling 93 breast cancer-associated miRNA genes leads to the identification of two functional promoters of the Hsa-mir-200b cluster, methylation of which is associated with metastasis or hormone receptor status in advanced breast cancer. Oncogene.

[21] Wang Z, Zang C, Cui K, Schones DE, Barski A, Peng W, Zhao K (2009). Genome-wide mapping of HATs and HDACs reveals distinct functions in active and inactive genes. Cell.

[22] Chen MC, Chen CH, Chuang HC, Kulp SK, Teng CM, Chen CS (2011). Novel mechanism by which histone deacetylase inhibitors facilitate topoisomerase IIalpha degradation in hepatocellular carcinoma cells. Hepatology.

[23] Sampath D, Liu C, Vasan K, Sulda M, Puduvalli VK, Wierda WG, Keating MJ (2012). Histone deacetylases mediate the silencing of miR-15a, miR-16, and miR-29b in chronic lymphocytic leukemia. Blood.

[24] Wendtner CM (2012). Cocktail of eternity: HDAC meets miR. Blood.

[25] Sikandar S, Dizon D, Shen X, Li Z, Besterman J, Lipkin SM (2010). The class I HDAC inhibitor MGCD0103 induces cell cycle arrest and apoptosis in colon cancer initiating cells by upregulating Dickkopf-1 and non-canonical Wnt signaling. Oncotarget.

[26] Lodrini M, Oehme I, Schroeder C, Milde T, Schier MC, Kopp-Schneider A, Schulte JH, Fischer M, De Preter K, Pattyn F, Castoldi M, Muckenthaler MU, Kulozik AE, Westermann F, Witt O, Deubzer HE (2013). MYCN and HDAC2 cooperate to repress miR-183 signaling in neuroblastoma. Nucleic Acids Res.

[27] Buurman R, Gurlevik E, Schaffer V, Eilers M, Sandbothe M, Kreipe H, Wilkens L, Schlegelberger B, Kuhnel F, Skawran B (2012). Histone deacetylases activate hepatocyte growth factor signaling by repressing microRNA-449 in hepatocellular carcinoma cells. Gastroenterology.

[28] Yuan JH, Yang F, Chen BF, Lu Z, Huo XS, Zhou WP, Wang F, Sun SH (2011). The histone deacetylase 4/SP1/microrna-200a regulatory network contributes to aberrant histone acetylation in hepatocellular carcinoma. Hepatology.

[29] He L, Yang H, Ma Y, Pledger WJ, Cress WD, Cheng JQ (2008). Identification of Aurora-A as a direct target of E2F3 during G2/M cell cycle progression. J Biol Chem.

[30] Jiang Y, Saavedra HI, Holloway MP, Leone G, Altura RA (2004). Aberrant regulation of survivin by the RB/E2F family of proteins. J Biol Chem.

[31] Druz A, Betenbaugh M, Shiloach J (2012). Glucose depletion activates mmu-miR-466h-5p expression through oxidative stress and inhibition of histone deacetylation. Nucleic Acids Res.

[32] Jayathilaka N, Han A, Gaffney KJ, Dey R, Jarusiewicz JA, Noridomi K, Philips MA, Lei X, He J, Ye J, Gao T, Petasis NA, Chen L (2012). Inhibition of the function of class IIa HDACs by blocking their interaction with MEF2. Nucleic Acids Res.

[33] Rodriguez-Paredes M, Esteller M (2011). Cancer epigenetics reaches mainstream oncology. Nat Med.

[34] Wang X, Yan Z, Fulciniti M, Li Y, Gkotzamanidou M, Amin SB, Shah PK, Zhang Y, Munshi NC, Li C (2014). Transcription factor-pathway coexpression analysis reveals cooperation between SP1 and ESR1 on dysregulating cell cycle arrest in non-hyperdiploid multiple myeloma. Leukemia.

[35] Hirose T, Horvitz HR (2013). An Sp1 transcription factor coordinates caspase-dependent and -independent apoptotic pathways. Nature.

[36] Fulciniti M, Amin S, Nanjappa P, Rodig S, Prabhala R, Li C, Minvielle S, Tai YT, Tassone P, Avet-Loiseau H, Hideshima T, Anderson KC, Munshi NC (2011). Significant biological role of sp1 transactivation in multiple myeloma. Clin Cancer Res.

[37] Kou XX, Hao T, Meng Z, Zhou YH, Gan YH (2013). Acetylated Sp1 inhibits PTEN expression through binding to PTEN core promoter and recruitment of HDAC1 and promotes cancer cell migration and invasion. Carcinogenesis.

[38] Mottet D, Pirotte S, Lamour V, Hagedorn M, Javerzat S, Bikfalvi A, Bellahcene A, Verdin E, Castronovo V (2009). HDAC4 represses p21(WAF1/Cip1) expression in human cancer cells through a Sp1-dependent, p53-independent mechanism. Oncogene.

[39] Pulikkan JA, Peramangalam PS, Dengler V, Ho PA, Preudhomme C, Meshinchi S, Christopeit M, Nibourel O, Muller-Tidow C, Bohlander SK, Tenen DG, Behre G (2010). C/EBPalpha regulated microRNA-34a targets E2F3 during granulopoiesis and is down-regulated in AML with CEBPA mutations. Blood.

[40] Lui JC, Baron J (2013). Evidence that Igf2 down-regulation in postnatal tissues and up-regulation in malignancies is driven by transcription factor E2f3. Proc Natl Acad Sci U S A.

[41] Bilke S, Schwentner R, Yang F, Kauer M, Jug G, Walker RL, Davis S, Zhu YJ, Pineda M, Meltzer PS, Kovar H (2013). Oncogenic ETS fusions deregulate E2F3 target genes in Ewing sarcoma and prostate cancer. Genome Res.

[42] Shen H, Morrison CD, Zhang J, Underwood W, Yang N, Frangou C, Eng K, Head K, Bollag RJ, Kavuri SK, Rojiani AM, Li Y, Yan L, Hill A, Woloszynska-Read A, Wang J (2013). 6p22. 3 amplification as a biomarker and potential therapeutic target of advanced stage bladder cancer. Oncotarget.

[43] You JS, Kang JK, Lee EK, Lee JC, Lee SH, Jeon YJ, Koh DH, Ahn SH, Seo DW, Lee HY, Cho EJ, Han JW (2008). Histone deacetylase inhibitor apicidin downregulates DNA methyltransferase 1 expression and induces repressive histone modifications via recruitment of corepressor complex to promoter region in human cervix cancer cells. Oncogene.

[44] Drummond DC, Noble CO, Kirpotin DB, Guo Z, Scott GK, Benz CC (2005). Clinical development of histone deacetylase inhibitors as anticancer agents. Annu Rev Pharmacol Toxicol.

[45] Wu CC, Yang TY, Yu CT, Phan L, Ivan C, Sood AK, Hsu SL, Lee MH (2012). p53 negatively regulates Aurora A via both transcriptional and posttranslational regulation. Cell Cycle.

[46] Wang R, Huang J, Feng B, De W, Chen L (2012). Identification of ING4 (inhibitor of growth 4) as a modulator of docetaxel sensitivity in human lung adenocarcinoma. Mol Med.

[47] Wang R, Wang ZX, Yang JS, Pan X, De W, Chen LB (2011). MicroRNA-451 functions as a tumor suppressor in human non-small cell lung cancer by targeting ras-related protein 14 (RAB14). Oncogene.

[48] Bracken CP, Gregory PA, Kolesnikoff N, Bert AG, Wang J, Shannon MF, Goodall GJ (2008). A double-negative feedback loop between ZEB1-SIP1 and the microRNA-200 family regulates epithelial-mesenchymal transition. Cancer Res.

