# Supplementary material for: HDAC 1/4-mediated silencing of microRNA-200b promotes chemoresistance in human lung adenocarcinoma cells
Source: Oncotarget. 2014 May 7;5(10):3333–49. doi: 10.18632/oncotarget.1948 (PMC4102813; doi:10.18632/oncotarget.1948)
Supplement: Supplementary file 1 [file oncotarget-05-3333-s001.pdf]

HDAC 1/4-mediated silencing of microRNA-200b promotes chemoresistance in human lung adenocarcinoma cells

Supplementary Material

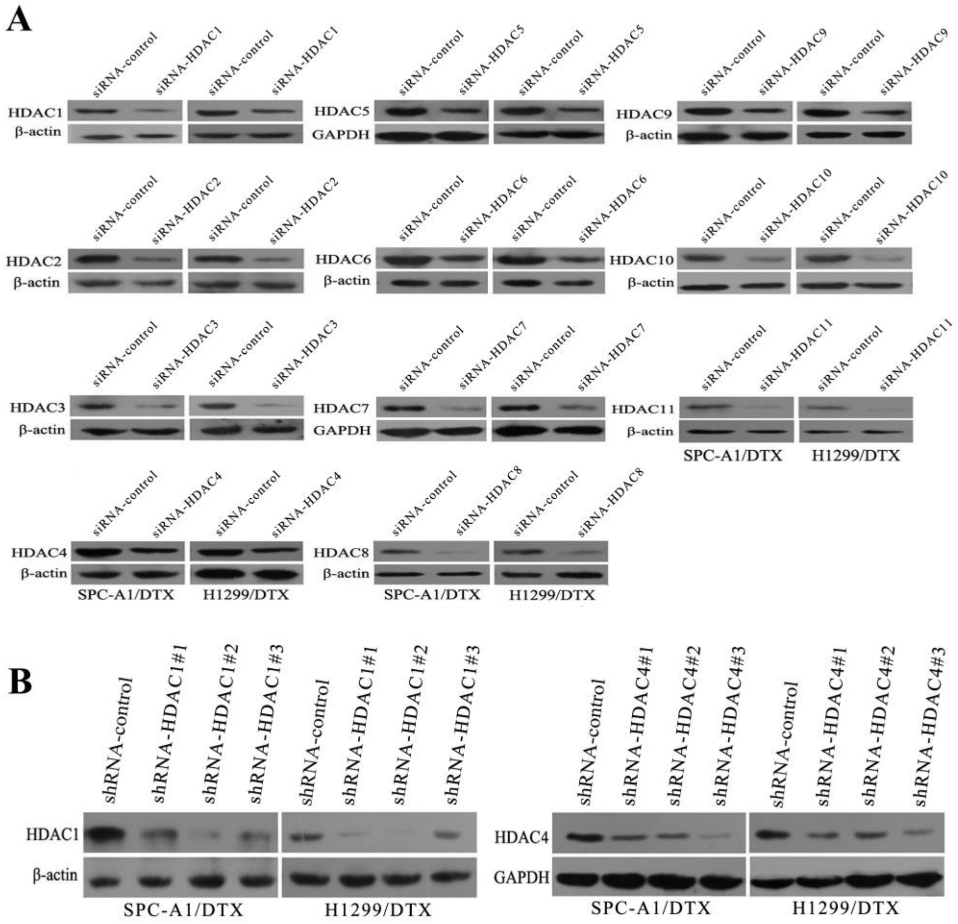

Supplementary Figure 1: Inhibitory efficiency of siRNA-HDACs or shRNA-HDAC1/4 in H1299/DTX and SPC-A1/DTX cells after transfection with the indicated vectors.

(A) Protein levels of the indicated HDACs after transfection with siRNA-HDACs or control vectors into H1299/DTX or SPC-A1/DTX cells. β-actin was used as an internal control. (B) Protein levels of HDAC1/4 as determined by western blot in docetaxel-resistant LAD cells transfected with sh-control, sh-HDAC1(#1, 2or 3) or sh-HDAC4 (#1, 2 or 3). β-actin or GAPDH was used as an internal control.

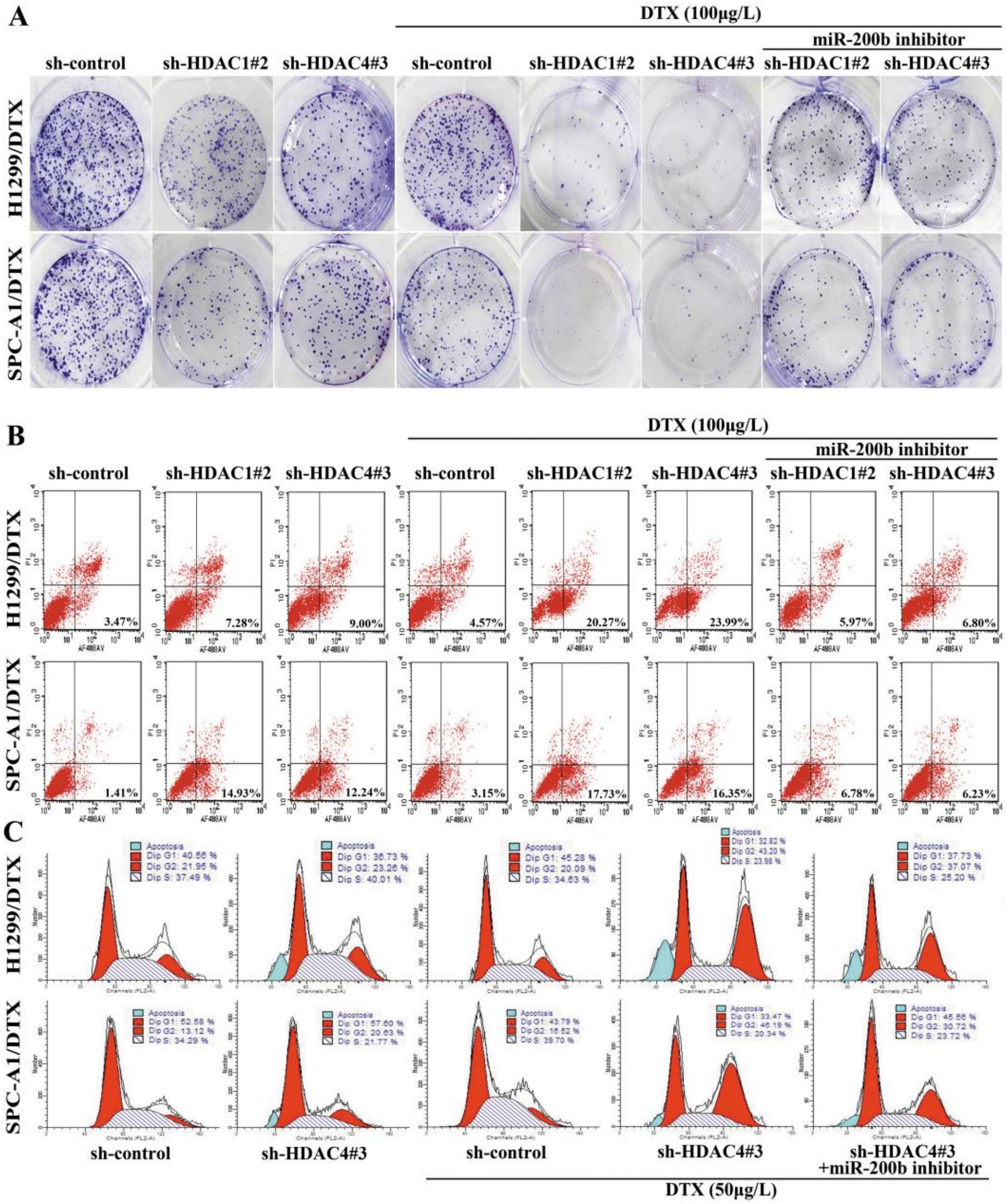

Supplementary Figure 2: Representative images of colony formation, apoptosis and cell cycle in docetaxel-resistant LAD cells after inhibition of HDAC1 and HDAC4. (A) The colony formation assay was performed as described in Methods. The number of colonies was counted and compared. (B) Flow cytometric analysis of apoptosis revealed that downregulation of HDAC1 and HDAC4 significantly elevated early-stage apoptosis levels of docetaxel-resistant LAD cells partially in a miR-200b-dependent manner. (C) Cell cycle analysis as measured by flow cytometry shown that downregulation of HDAC4 induced cell cycle arrest in G_2/M phase, partially in a miR-200b-dependent manner.

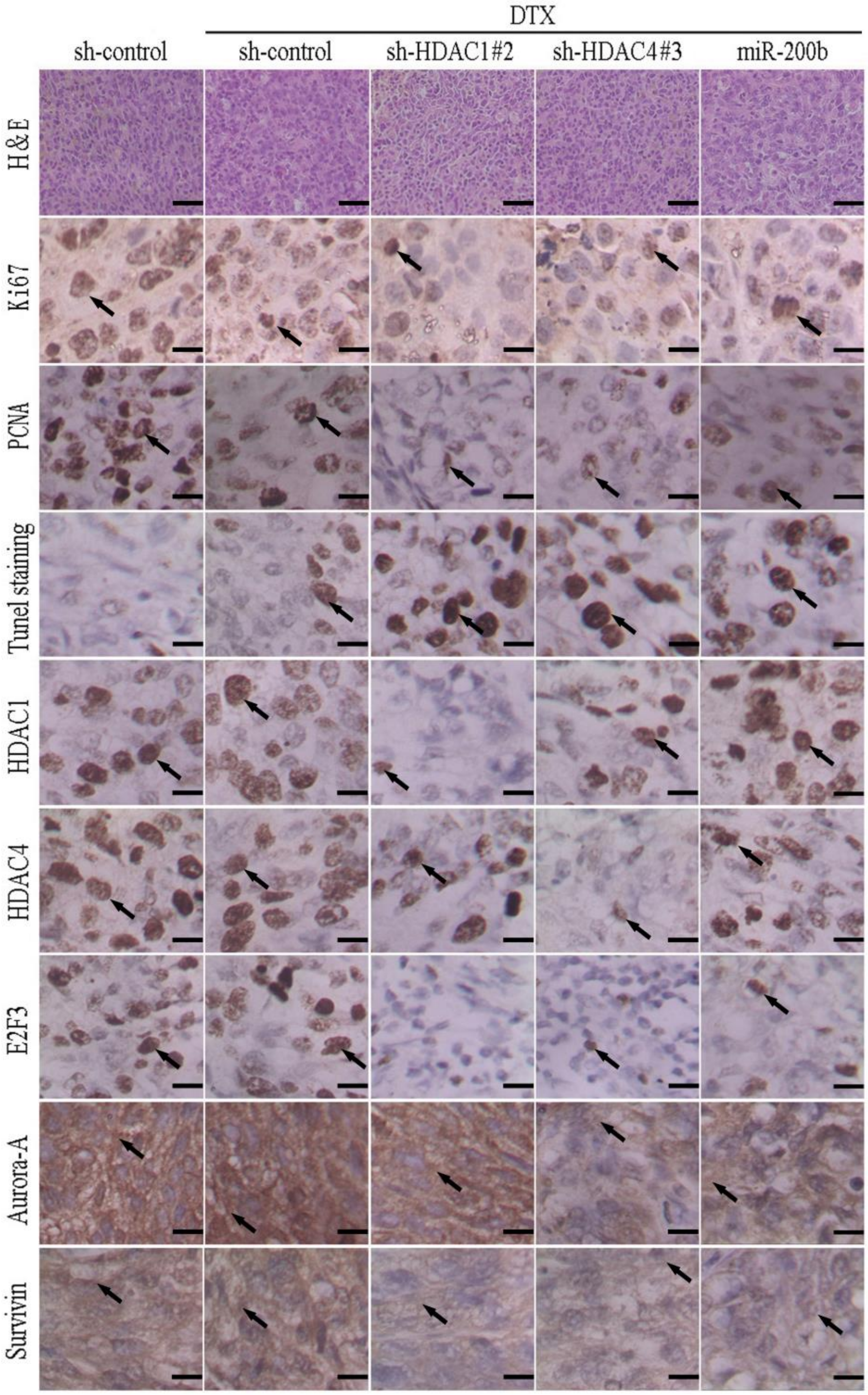

Supplement Figure 3: Hematoxylin and eosin (H&E) and immunohistochemistry staining in the xenograft transplantation experiment. H&E staining, proliferating cell nuclear antigen (PCNA) staining, TUNEL staining and immunohistochemistry staining of HDAC1, HDAC4, E2F3, survivin and Aurora-A were performed in tumors 6 weeks after inoculation. The black arrows were considered as positive areas. Scale bar, 50 μm.

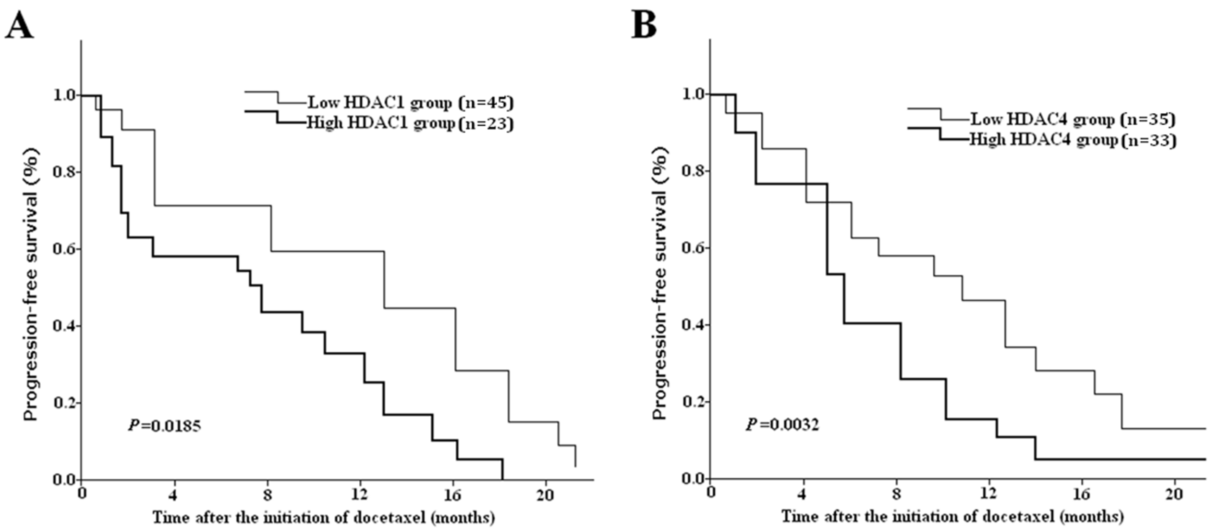

Supplementary Figure 4: Correlations of HDAC1/4 expression with survival of LAD patients who received docetaxel-based chemotherapies. (A) Statistical analysis of progression-free survival (PFS) of LAD patients according to HDAC1 mRNA expression level in tumor tissues. (B) Statistical analysis of PFS of LAD patients according to the level of HDAC4 mRNA expression in tumor tissues. The *P*-value was determined with the log-rank test.
