# Supplementary material for: HDAC 1/4-mediated silencing of microRNA-200b promotes chemoresistance in human lung adenocarcinoma cells
Source: Oncotarget. 2014 May 7;5(10):3333–49. doi: 10.18632/oncotarget.1948 (PMC4102813; doi:10.18632/oncotarget.1948)
Supplement: Supplementary file 2 [file oncotarget-05-3333-s002.pdf]

HDAC 1/4-mediated silencing of microRNA-200b promotes chemoresistance in human lung adenocarcinoma cells

Supplementary Material

Supplementary Table 1: Primers for promoter experiments

name			primer sequences
miR200b Promoter-1	(KpnI)	F	5'-CGGGGTACCCAGAGGTGGAGAGGCGAGAG-3'
	(HindIII)	R	5'-CCCAAGCTTGGGGCCTCGGGAGGGAAGAGC-3'
miR200b Promoter-2	(KpnI)	F	5'-CGGGGTACCTTTACAGCCCGGATCACTGG-3'
	(HindIII)	R	5'-CCCAAGCTTCGCTTTCTTGTCAACCGTCG-3'
miR200b promoter-1,Sp1-1 mut	(KpnI)	F	5'-CGGCAAGGTGGGTTCGGGACGGAGTCTGCG-3'
	(HindIII)	R	5'-CGTCCCGAACCCACCTTGCCGCCCACCGAG-3'
miR200b promoter-1,Sp1-2 mut	(KpnI)	F	5'-CCTGTGTGGTTCGGGGAGCACTGCTCCTTG-3'
	(HindIII)	R	5'-TGCTCCCCGAACCACACAGGTGCGAGCTCC-3'
miR200b promoter-2, Sp1 mut	(KpnI)	F	5'-TGTTCTCTGTGGTTCGGGTGGACGTGGCCCGGAC-3'
	(HindIII)	R	5'-TCCACCCGAACCACAGAGAACACACCAGCTCCT -3'
survivin promoter	(KpnI)	F	5'-CGGGGTACCGACTACAACTCCCGGCACAC -3'
	(HindIII)	R	5'-CCCAAGCTTGCCGCCGCCGCCACCTCTGC -3'
survivin promoter, E2F3 mut	(KpnI)	F	5'-GCCCCGCGGCTTGCCATTAACCGCCAGATTTGA -3'
	(HindIII)	R	5'-CGGTTAATGGCAAGCCGCGGGGCATGTCGGGAG -3'
aurora-A promoter **[1]**	(MluI)	F	5'-CGACGCGTTGGCTCCACCACTTCCGG -3'
	(BglII)	R	5'- GAAGATCTCTCTAGCTGTAATAAGTAAC -3'
aurora-A promoter E2F3-1mut **[1]**	(MluI)	F	5'-GGCTCCACCACTTCATGGTTCTTAGGGAGC -3'
	(BglII)	R	5'-GCTCCCTAAGAACCATGAAGTGGTGGAGCC -3'
aurora-A promoter E2F3-2mut **[1]**	(MluI)	F	5'-GAGCAAGTCGCCTGCATGCGGTGTGCGCCCTT -3'
	(BglII)	R	5'-AAGGGCGCACACCGCATGCAGGCGACTTGCTC -3'

Supplementary Table 2: Primers for sh-RNA experiments

name		primer sequences
sh-HDAC1 #1	F	5'-CACCGCTCCATCCGTCCAGATAACATTCAAGAGATGTTATCTGGACGGATGGAGCTTTTTTG-3'
	R	5'-GATCCAAAAAAGCTCCATCCGTCCAGATAACATCTCTTGAATGTTATCTGGACGGATGGAGC -3'
sh-HDAC1 #2	F	5'-CACCGGAGAGTACTTCCCAGGAACTTTCAAGAGAAGTTCCTGGGAAGTACTCTCCTTTTTTG-3
	R	5'-GATCCAAAAAAGGAGAGTACTTCCCAGGAACTTCTCTTGAAAGTTCCTGGGAAGTACTCTCC-3'
sh-HDAC1 #3	F	5'-CACCGGTGAGGACTGTCCAGTATTCTTCAAGAGAGAATACTGGACAGTCCTCACCTTTTTTG-3'
	R	5'-GATCCAAAAAAGGTGAGGACTGTCCAGTATTCTCTCTTGAAGAATACTGGACAGTCCTCACC-3'

sh-HDAC4 #1	F	5’-CACCGGAGATGCTGGCCATGAAGCATTCAAGAGATGCTTCATGGCCAGCATCTCCTTTTTTG-3’
	R	5’-GATCCAAAAAAGGAGATGCTGGCCATGAAGCATCTCTTGAATGCTTCATGGCCAGCATCTCC-3’
sh-HDAC4 #2	F	5’-CACCGCGTGGGTTTCAACGTCAACATTCAAGAGATGTTGACGTTGAAACCCACGCTTTTTTG-3’
	R	5’-GATCCAAAAAAGCGTGGGTTTCAACGTCAACATCTCTTGAATGTTGACGTTGAAACCCACGC-3’
sh-HDAC4 #3	F	5’-CACCGCAGCAGCATCAGCAGTTTCTTTCAAGAGAAGAAACTGCTGATGCTGCTGCTTTTTTG-3’
	R	5’-GATCCAAAAAAGCAGCAGCATCAGCAGTTTCTTCTCTTGAAAGAAACTGCTGATGCTGCTGC-3’
sh-control	F	5’-CACCGTTCTCCGAACGTGTCACGTTTCAAGAGAACGTGACACGTTCGGAGAATTTTTTG-3’
	R	5’-GATCCAAAAAATTCTCCGAACGTGTCACGTTCTCTTGAAACGTGACACGTTCGGAGAAC -3’

Supplementary Table 3: Primers for real-time quantitative PCR

name		primer sequences
miR-200b [2]	Stem-loop	5’-GTCGTATCCAGTGCAGGGTCCGAGGTATTCGCACTGGATACGACTCATCA-3'
	F	5'-GTGGAGGGTCCGAGGTATTC-3'
	R	5'-CGTAATACTGCCTGGTAATGATG-3'
U6	F	5’-CTCGCTTCGGCAGCACA-3'
	R	5’-AACGCTTCACGAATTTGCGT-3'
HDAC1	F	5’-TAACCTGCCTATGCTGATGCT-3'
	R	5'-CTCATTCGTGTTCTGGTTAGTCATA-3'
HDAC4	F	5'-AAGAACAAGGAGAAGGGCAAAG-3'
	R	5'-TGGAGAACTCTGGTCAAGGGA-3'
E2F3	F	5’-AGAAAGCGGTCATCAGTACCT-3’ (PrimerBank ID340523139c1)
	R	5’-TGGACTTCGTAGTGCAGCTCT-3’ (PrimerBank ID340523139c1)
Aurora-A	F	5’-AGCTGGAGAGATGCTGGT-3’
	R	5’-ACTGGTAGGATTGCGGTGGA-3’
Survivin	F	5’-CCACTGAGAACGAGCCAGAC-3’
	R	5’-TTTGCAACTCAAATCTTTTGACA-3’
GAPDH	F	5'-TGGGTGTGAACCATGAGAAGT-3'
	R	5'-TGAGTCCTTCCACGATACCAA-3'

Supplementary Table 4: Primers for Chip-PCR

name		primer sequences
miR-200b P1-1	R	5’-CCTACCCCTGCCCATGTCG-3’
	F	5’-TGGCCAATCTCCGCTCCTG-3’
miR-200b P1-2	R	5’-GGGCCAGGAGCGGAGATTG-3’
	F	5’-CGAGGGCGACGGGACAATG-3’
miR-200b P1	R	5’-TTTTGTTCAACCTCGGTGGGC-3’
	F	5’-GGCTCGCCTTACAAGGAGCAGT-3’
miR-200b P2	R	5’-GCGAAACGAGTTTGTCAGAACA-3’
	F	5’-GGCGTGGATTTGCAGCCCTC-3’
P21 [3]	R	5’-GCACCAACGCAGGCGAGGGACT-3’
	F	5’-GCAGCTGAGCCTGGCCGAGTTC-3’
survivin	R	5’-GACTACAACTCCCGGCACAC-3’
	F	5’-CGATTCAAATCTGGCGGTTA-3’
aurora-A [1]	R	5’-CGACGCGTTGGCTCCACCACTTCCGG-3’
	F	5’-CCAGGAGCTCAGCCGTTAGAATTCAAAGG-3’

Supplementary Table 5: Correlations of HDAC1/4 expression with clinicopathological variables of LAD patients

	HDAC1 expression			HDAC4 expression		
Variables	High (n=23)	Low (n=45)	*P*-value	High (n=35)	Low (n=33)	*P*-value
Gender			0.159			0.347
Female	16	24		12	15	
Male	7	21		23	18	
Age (years)			0.442			0.598
<60	10	18		16	13	
≥60	13	27		19	20	
Differentiation			0.444			0.493
Well+Moderate	8	20		14	15	
Poor	15	25		21	16	
Clinical stage			0.003*			0.004*
IIIB	6	29		11	22	
IV	17	16		24	11	
Tumor response			0.030*			0.001*

CR+PR	5	22	28	14
SD+PD	18	23	7	19

*P<0.05.

REFERENCES

1. He L, Yang H, Ma Y, Pledger WJ, Cress WD, and Cheng JQ. Identification of Aurora-A as a direct target of E2F3 during G2/M cell cycle progression. J Biol Chem. 2008; 283(45):31012-31020.
2. Yuan JH, Yang F, Chen BF, Lu Z, Huo XS, Zhou WP, Wang F, and Sun SH. The histone deacetylase 4/SP1/microrna-200a regulatory network contributes to aberrant histone acetylation in hepatocellular carcinoma. Hepatology. 2011; 54(6):2025-2035.
3. Mottet D, Pirotte S, Lamour V, Hagedorn M, Javerzat S, Bikfalvi A, Bellahcene A, Verdin E, and Castronovo V. HDAC4 represses p21(WAF1/Cip1) expression in human cancer cells through a Sp1-dependent, p53-independent mechanism. Oncogene. 2009; 28(2):243-256.
